# Closing the Loop: High‐Precision 3D Photofabrication in Living Tissues

**DOI:** 10.1002/advs.77853

**Published:** 2026-09-27

**Authors:** Amirbahador Zeynali, Mangirdas Malinauskas, Giuseppe Chirico

**Affiliations:** ^1^ Institute of Biomaterials and Biomolecular Systems University of Stuttgart Stuttgart Germany; ^2^ Laser Research Center Faculty of Physics Vilnius University Vilnius Lithuania; ^3^ Department of Physics “G. Occhialini” University of Milano‐Bicocca Milan Italy

**Keywords:** biophotonic sensors, closed‐loop control, intravital photofabrication, two‐photon polymerization

## Abstract

Multiphoton absorption enables three‐dimensional fabrication of photoresists ranging from ceramics to hydrogels and has recently been extended to the writing of microstructures within the dermis, muscle, and brain of living animals. However, an intrinsic difficulty prevents its widespread application in vivo. Absorption, cure kinetics, heat accumulation, and matrix stiffness each vary steeply across neighboring micro‐environments and across intervals spanning a single pulse to several minutes. Therefore, any pre‐selected exposure drifts out of the safe and effective window as fabrication proceeds. Rigorous feedback control should ideally act simultaneously at three scales: between pulses to bound heating (microseconds), within each voxel to confirm cure and stiffness (milliseconds), and across the finished volume to verify geometry (seconds). Phase‐resolved optical coherence tomography and luminescent thermometry already satisfy these demands, with photoacoustic, Brillouin, and diamond‐defect techniques reaching further into depth, contrast, and sensitivity. We analyze critically how this technology supports predictive controllers at the three scales that learn tissue behavior on the fly, turning these readings into adaptive feedforward with supervisory feedback. We synthesize this toolkit into a single perspective toward adaptive intravital light interventions.

## Graphical abstract

Writing 3D microstructures inside living tissue demands more than laser access; It demands information. Hierarchical sensing captures thermal and mechanical states across pulse‐train, voxel, and structure timescales, feeding a controller that steers the laser in real time. Closing this loop turns open‐loop guesswork into adaptive, feedback‐guided intravital photofabrication.

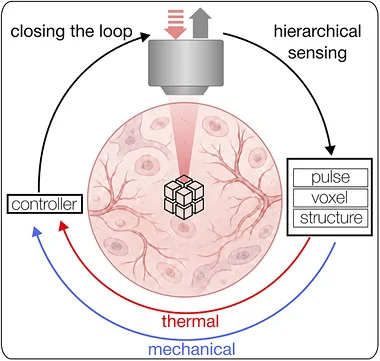

## 1 Introduction

### 1.1 The Promise and Challenge of Direct In Vivo Fabrication

Light‐driven three‐dimensional fabrication has progressed from cuvettes and bulk hydrogels to direct deposition inside living tissue. Recent demonstrations [1, 2, 3, 4] have written femtoliter‐scale structures within mouse dermis, skeletal muscle, and brain, establishing intravital photofabrication as a credible route to repair of structures inaccessible to surgery or too fragile to survive ex vivo assembly and reimplantation. Three conditions must be met for this approach to mature into a clinical modality: (i) optical access to the volume through near‐infrared (NIR) transparency windows or endoscopic delivery, (ii) volumetric confinement of the photochemical reaction to the focal voxel to avoid collateral damage along the beam path and (iii) continuous monitoring of the thermal, mechanical and chemical load onto the surrounding tissue. The nonlinear origin of multiphoton polymerization (MPP) circumvents the first two (**Box 1**), the third has, to date, remained unresolved and is the subject of this perspective.

**BOX 1: Multiphoton Quantitative Framework**

**Photophysical Basis**

Two‐photon absorption (TPA) requires the near‐simultaneous arrival of two photons within ∼10^−15^ s. This probability is governed by the two‐photon cross‐section *σ*_2_, measured in Göppert‐Mayer (GM) units:

*σ*_2_ ∼ 1–100 GM where 1 GM = 10^−50^ cm^4^·s·photon^−1^

The cross‐sections relevant to intravital intervention are modest from endogenous chromophores and water‐soluble biocompatible initiators [5], whereas dedicated donor‐acceptor initiators engineered for ex vivo fabrication reach 10^2^–10^3^ GM [6].

**Excitation Rate**

The two‐photon excitation rate per molecule [7]:

$$dN/dt=\sigma_{2}{\left(I/\hbar \omega \right)}^{2}=\sigma_{2}I^{2}/{\left(\hbar \omega \right)}^{2}$$

The $I^{2}$ **dependence** confines excitation to the focal volume where intensity is maximal.

**Threshold Behavior**

Polymerization initiates when radical generation rate exceeds oxygen inhibition and diffusive loss:

$$I^{2}>I_{\text{th}}^{2}\propto \frac{k_{O_{2}}\left[O_{2}\right]+k_{d}}{\sigma_{2}\Phi_{R}}$$

where $\Phi_{R}$ is the radical quantum yield. This threshold creates a well‐defined, spatially confined boundary between polymerized and unpolymerized regions. The rates $k_{O_{2}}$ and $k_{d}$ describe the rates of Oxygen inhibition through radical scavenging and the radical loss from the focal volume due to diffusion and termination processes, respectively.

**Voxel Dimensions**

The polymerized voxel is ellipsoidal, with dimensions set by the point spread function:

**Table:** 

Axis	Formulation	Validity
Lateral (xy)	$\approx \frac{0.320\lambda }{\sqrt{2}NA}$	*NA*≤0.7
Lateral (xy)	$\approx \frac{0.325\lambda }{\sqrt{2}NA^{0.91}}$	*NA*>0.7
Axial (z)	$\approx \frac{0.532\lambda }{\sqrt{2}\left(n-\sqrt{n^{2}-NA^{2}}\right)}$	all *NA*

These expressions give the optical (microscopy resolution) envelope. The actual polymerized volume is threshold‐ and dose‐dependent and is generally smaller, since polymerization occurs only where the radical density exceeds the gelation threshold.

**Typical TPP Parameters**

**Table:** 

Parameter	Value	Rationale
Wavelength	780–820 nm	NIR window: minimal H_2_O absorption
Pulse duration	80–150 fs	High peak power, low average power
Repetition rate	80 MHz	Thermal relaxation between pulses
Pulse energy	0.1–1 nJ	Below tissue damage threshold
Peak intensity	10^1^ ^1^–10^1^ ^2^ W/cm^2^	Above polymerization threshold
Accumulated dose	0.1 – 10 nJ	Depends on scanning speed / dwell time
Average power	10–50 mW	Limits bulk heating
Focal volume	∼0.1 µm^3^	∼0.1 fL

**Depth Limitation**

Effective penetration depth in tissue [8, 9]:

$$z_{\mathrm{eff}}=\left(1/{\mu^{'}}_{s}\right)\ln\left(I_{0}/I_{\mathrm{th}}\right)$$

For soft tissue (${\mu^{'}}_{s}$≈ 1.3‐1.4 mm^−1^), practical depths reach **0.5–2 mm** before scattering degrades focal quality below threshold.

The practical depth limit hinges on the ratio $I_{0}/I_{\mathrm{th}}$, where $I_{0}$ must remain below the maximum permissible exposure (MPE) for skin while exceeding the polymerization threshold $I_{\mathrm{th}}$. For femtosecond‐pulsed TPP, this is achievable: tissue damage is governed by average power (~ 200 ‐ 400 mW · cm^−2^ at 800 nm), whereas polymerization depends on peak intensity (0.1 ‐ 1 TW · cm^−2^). Shifitng to 1300 nm reduces scattering (${\mu^{'}}_{s}$ ≈ 1 mm^−1^) and improves this margin, though water absorption becomes relevant.

**Maximum permissible exposure (skin)**

One of the most difficult problems in developing the exposure limits concerns repetitive‐pulse exposure where the individual pulse duration is less than about 10 µ*s*,  as is the case in multiphoton fabrication. Between 700 and 1400 nm [10], exposure limits for both eye and skin include a constant spectral correction factor $C_{A}=10^{0.002(\frac{\lambda }{1nm}-700)}\;$ and scales as $EL=EL_{min}{\left(\frac{\tau_{E}}{1s}\right)}^{0.25}C_{A},$ where $\tau_{E}=\frac{w_{0}}{v_{s}}$ is the effective exposure time over the beam waist whose size is $w_{0}$ and $EL_{\min}$ =  11 kJ · m^−2^ .

The non‐linear damage mechanisms which at lower intensities (≃ 0.1 TW · cm^−2^) is at the very basis of photopolymerization, is additive to a good approximation though it greatly depends on the heat dissipation rate. For thermal injury (due mainly to linear absorption of water), when the energy is delivered during the thermal confinement time, e.g., over a duration in which there is no significant heat dissipation during the exposure, the total cumulative dose also determines the thermally induced biological effect. As an example for skin, if we polymerize with a focused laser beam (*NA* = 0.5, λ  =  800 *nm*) at 300 µ*m* depth beneath the skin surface, the limit dose EL ≃ 8 kJ ·  m^−2^ =  0.8 J · cm^−2^. This value can be obtained when dynamically fabricating with a scanning speed $v_{s}>20\;\mu m\cdot s^{-1}$.

The defining feature of MPP is that excitation probability scales nonlinearly with local intensity (∝ $I^{N}$ with *N*  =  2 for the dominant two‐photon implementation, TPP). Photochemistry initiates only where focal intensity exceeds a distinct threshold, restricting polymerization to a femtoliter focal volume while tissue along the beam path remains essentially inert [11]. Three‐dimensional selectivity is therefore achieved without physical contact or layer‐by‐layer deposition, with the further advantage that femtosecond pulses shift the operating regime from a wavelength‐determined response to an intensity‐determined one [12], enabling crosslinking across the tissue transparency windows and, in some formulations, without exogenous photoinitiator [12, 13, 14].

The clinical scope of intravital photofabrication extends to procedures that are, as of today, surgically inaccessible like reconstructing spinal cord architecture without laminectomy, reinforcing degenerating cardiac valves during continuous perfusion, or bridging neural gaps in the cortex through minimally invasive optical ports. Yet realizing this potential requires solving a problem that ex vivo TPP does not face. Biological tissue is heterogeneous, dynamic, and optically thick. The same scattering and absorption that limit the depth of the optical access also perturb the focal intensity distribution, shifting the local balance between successful polymerization and thermal damage in a manner that cannot be predicted from preoperative imaging alone. Two complementary strategies have emerged to mitigate this constraint: (i) wavefront shaping methods that partially correct focal distortion in real time [15, 16], and (ii) in vivo tissue‐clearing agents that transiently reduce scattering by refractive index matching at the tissue level [17, 18]. However, these corrections address only the optical part of the problem.

Two terminological points warrant clarification. (i) We use multiphoton polymerization for the general N‐photon phenomenon and reserve two‐photon polymerization for the specific implementation that underlies essentially all reported in vivo demonstrations to date. (ii) We further distinguish laser‐based intravital photofabrication, the high‐resolution multiphoton process discussed throughout this manuscript, from bioprinting in its conventional usage which refers to extrusion‐based bioprinting of hydrogels and cell suspensions at millimeter resolution [19].

MPP allows sub‐diffraction writing. Because polymerization proceeds only where the intensity exceeds a threshold, the reaction is confined to the center of the focal volume and the smallest features can fall below the diffraction limit. The solidified feature need not coincide with the optical voxel, however. Depending on the resin chemistry, radical and thermal diffusion can extend polymerization beyond the illuminated region, while inhibitors or quenchers can sharpen the threshold and suppress it [5, 20], so the polymerized volume can be either larger or smaller than the voxel. The dimensions [21] of this optical voxel (1/e radius of the squared intensity $I_{PSF}^{2}$ profile of a focused Gaussian beam) scale with wavelength and numerical aperture (*NA*), with closed‐form expression given in **Box 1**. These spatial dimensions, however, set only the geometric envelope within which polymerization occurs. The extent and completeness of polymerization within that envelope are governed by the chemistry of radical generation, which separates into different time scales. The instantaneous absorption rate is nonlinear in I and sets a confined spatial threshold for crosslinking. The cumulative absorbed energy, by contrast, integrates this rate over the local exposure time and it scales linearly with dwell time *τ* or, equivalently, inversely with scan velocity $v_{s}$. When intensity exceeds the polymerization threshold, the accumulated dose *D_acc_* determines the extent of conversion within the voxel, and consequently the mechanical properties of the printed structure [22]. This separation provides an experimentally useful degree of freedom. High I with low *D_acc_* produces confined but partial conversion, whereas longer dwell near threshold drives conversion toward completion at the cost of voxel growth, as accumulated dose pushes the wings of the intensity distribution above the polymerization threshold. This resolution‐vs.‐conversion trade‐off is intrinsic to the threshold mechanism. Ultrafast lasers enable multi‐photon polymerization via ∼TW · cm^−2^ intensities and therapeutic exposure doses, as validated on live cells and whole organisms [23, 24, 25].

**FIGURE 1:**
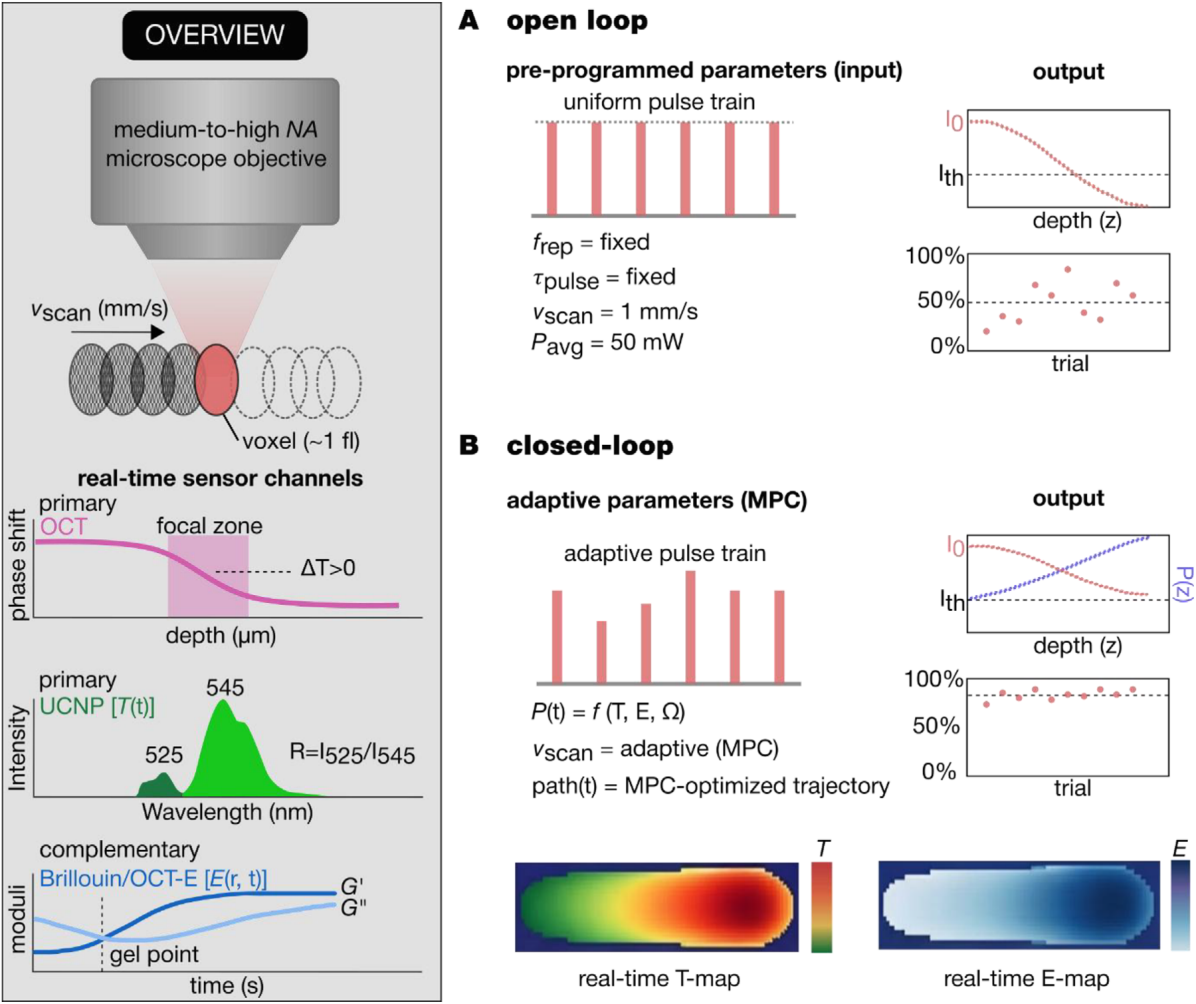
Open‐loop vs. closed‐loop intravital photofabrication. A femtosecond laser writes at the femtoliter voxel scale (V_voxel_ ∼ 1 fL) at scan velocity *v*_s_, with two primary sensing channels: multimodal OCT, which simultaneously extracts the thermo‐optic phase Δ*φ*(z,t) ∝ (d*n*/d*T*)·Δ*T* across the heated focal zone (shaded) and the elastographic strain field from a single backscattered signal, and UCNP ratiometric thermometry, exploiting the temperature dependence of Er^3^^+^ emission ratio *R* = *I*_5__2__5_/*I*_5__4__5_ that operate continuously alongside one or more complementary sensing channels selected by spatial scale and precision demand. Examples include Brillouin spectroscopy for label‐free mechanical readout across the gelation transition (gel point marked), and NV‐diamond thermometry for sub‐millikelvin temperature precision at the single‐voxel scale (A) Open‐loop operation: a uniform pulse train with fixed parameters is delivered blindly. The focal intensity *I*(z) decays with depth and crosses the polymerization threshold *I*_th_ stochastically, producing broadly scattered success rates across repeated trials. (B) Closed‐loop operation: a model predictive controller (MPC) adapts the laser power *P*(t), scan velocity, and trajectory in response to the sensor stream. The adaptive pulse train ramps *P*(z) (rising dashed blue curve) to compensate for tissue attenuation and keep *I*(z) near *I*_th_ throughout the depth range, while real‐time temperature (T‐map) and elastic‐modulus (E‐map) fields verify safe operation and reproducible outcomes (>90% across trials). MPC, model predictive control; UCNP, upconversion nanoparticle; OCT, optical coherence tomography.

The current use of 3D photo‐polymerization in intravital photofabrication is fundamentally “*blind*”. Demonstrations range from transcutaneous printing in live mice [3, 4], endoscopic fiber delivery [26, 27] and even intracellular fabrication [28]. All operate open‐loop, with laser parameters fixed a priori based on literature‐derived tissue properties. Miniaturizing the delivery optics addresses resolution, not awareness and control. A fiber at the tissue interface is no less blind to local conditions than a benchtop objective centimeter away. Even when these trials remain within the maximum permissible exposure limits for skin [29] in the near‐infrared window (**Box 1**), operating below the damage threshold does not by itself guarantee successful polymerization. Photodamage is not a sharp threshold but a graded, probabilistic process. A focused femtosecond exposure induces measurable phototoxicity well below ablation, with a damage probability that rises continuously with power, dwell time, and cumulative exposure [30]. Direct evidence for this comes from in situ cell encapsulation achieved through two‐photon polymerization, where functional structures are fabricated, but the cells within the exposed volume are damaged [31]. Similar patterns are found across various systems [32], including large variability of preclinical outcomes with thermal damage, incomplete polymerization, and poor host integration, all directly traceable to open‐loop operation [32] (Figure 1A). Translating intravital photofabrication into reproducible clinical use therefore requires sensors capable of resolving each relevant failure mode at its native spatiotemporal scale, and a control architecture that closes the loop between sensing and laser actuation (Figure 1B).

This architecture combines predictive feedforward and measurement‐driven feedback. The term “closed‐loop” is used here in this specific sense. A predictive model‐based layer anticipates the thermal response with measurement‐driven correction that acts on the sensed state. Closed‐loop can be reached only for the regimes in which control genuinely acts on measurements. As developed in §3.3 and §4.1, the pulse‐train level (microsecond timescale) is the regime in which this condition is met the least, because of the time response of sensors and of the photon statistics limitations.

### 1.2 The Challenge of Quantifying Heterogeneity

Tissue heterogeneity manifests simultaneously across physical (optical, thermal and mechanical) and biological (the metabolic state, cell‐cycle phase, fibrotic remodeling) axes. Each phenomenon varies over characteristic length scales, from nanometers to centimeters, and time scales, from microseconds to hours. These axes are not independent. They couple through the polymerization process itself and are treated separately in what follows only to clarify the sensing and control requirements each imposes.

Thermal conductivity, which governs heat dissipation from the printing voxel, varies substantially with local composition. Water‐rich parenchyma exhibits relatively high conductivity, whereas densely collagenous and lipid‐rich domains display lower values due to the poor thermal transport of protein fibers and insulating fatty phases [33, 34, 35, 36, 37]. Blood vessels introduce an additional layer of heterogeneity. At rest, its conductivity is comparable to surrounding tissue, but active perfusion produces a substantially higher effective conductivity through convective heat transport [38]. This compositional variability translates into a nearly one order of magnitude spread in thermal conductivity within a single organ, a heterogeneity that open‐loop fabrication cannot resolve.

Heat transport in heterogeneous, perfused tissue is described by the generalized Pennes bioheat equation [39, 40, 41]:

(1) $$\begin{array}{cc} \rho \left(r\right)c_{p}\;\frac{\partial T}{\partial t} & =\nabla \cdot \left[k\left(r\right)\cdot \nabla T\right]+\rho_{b}c_{b}\omega_{b}\left(r\right)\left(T_{a}-T\right)+Q_{\mathrm{met}}\left(r,t\right)\\ & \;+\mu_{a}\left(r\right)I\left(r,t\right)+\rho \left(r\right)\Delta H_{p}\frac{\partial D}{\partial t} \end{array}$$

with $Q_{\mathrm{met}}\left(r,t\right)$ the metabolic heat generation rate, space dependent density ρ(r), thermal conductivity k(r), specific heat $c_{p}$, blood perfusion term $\omega_{b}\left(r\right)$, and tissue absorption $\mu_{a}\left(r\right)$. The last term on the right‐hand side of Equation (1), $\rho \left(r\right)\Delta H_{p}\frac{\partial D}{\partial t}$, arises from the reaction enthalpy per unit converted mass,  $\Delta H_{p}$, of the exothermic polymerization. This term is proportional to the local conversion rate, $\frac{\partial D}{\partial t}$. Therefore, the kinetics of the temperature field carries information about how fast polymerization proceeds, complementing the modulus‐conversion link of Equation (3). To account for the heterogeneity and anisotropy of tissues, the thermal conductivity is better described by a tensor field. For example, in fibrous tissues such as myelinated white‐matter tracts or aligned muscle fibers, thermal conductivity is (modestly) anisotropic [42]. Heat diffuses preferentially along the fiber direction and the thermal load spreads asymmetrically, preferring specific pathways while sparing adjacent regions.

The mechanical axis spans the widest range, nearly seven orders of magnitude from soft brain tissue (∼ 0.5‐3 kPa) to mineralized bone (∼ 10 GPa) [43, 44]. Within a single organ, the variation could even evolve with disease state. In the kidney, bulk shear‐wave and magnetic resonance elastography [45, 46] consistently finds the cortex stiffer than the medulla, with values rising further under fibrotic remodeling [46, 47]. At the microscale, atomic force microscopy (AFM) can resolve stiffness variations between adjacent structures that bulk elastography averages out. For example, it can detect the cytoskeletal stiffening of renal tubular cells under fibrotic reaction [48, 49]. Since absolute values depend strongly on the AFM measurement modality, loading frequency and perfusion state we limit ourselves to a qualitative ordering (cortex > medulla > fibrotic > healthy) rather than quoting values.

The implication of heterogeneity for intravital photofabrication is conceptual. The mechanical compliance that a printed structure must match is not a fixed tissue property but a state variable that drifts as the host responds over hours to weeks. These mechanical variations critically affect printing outcomes. Photopolymerization may induce volumetric shrinkage of several percent to ∼20%, generating internal stresses whose magnitude depends on mechanical constraint from the surrounding environment [50]. In stiff environments, shrinkage under constraint generates tensile stresses that can reach hundreds of kPa to a few MPa. These values are sufficient to drive delamination, critically affecting the integration of newly fabricated structures with host tissue. In compliant environments, the same shrinkage is accommodated by deformation of the surrounding matrix without interfacial failure, but the printed structure itself may buckle or lose dimensional fidelity.

Optical heterogeneity is discrete rather than smooth. Plasma has negligible absorption [9] in the visible range (500 nm ‐ 600 nm), while red blood cells create absorption hot spots due to the high absorption of hemoglobin whose µ_*a*_ exceeds 100 cm^−1^ in the visible. The issue is mitigated in the NIR biological window (800 nm) where *µ*_a_ drops to approximately 1 − 5 cm^−1^ for hemoglobin, depending on the oxygenation state. The spatial distribution of the absorption hot spots follows the vascular architecture, built by capillaries that can be widely (20–50 µm apart) or densely (5 µm apart) spaced in basal or metabolically active organs like heart and brain [51]. Between vessels, cellular chromophores, mitochondrial cytochromes (*µ*_a_ =  0.1 − 1 cm^−1^), lipofuscin in aging cells (*µ*_a_ =  1 − 10 cm^−1^), and melanin in specialized cells (*µ*_a_ >100 cm^−1^), contribute baseline absorption [52, 53].

Tissue is also dynamic. Cardiac pulsation propagates pressure waves through soft tissues at 5–15 m·s^−1^ [54], driving cyclic changes in local blood volume that modulate tissue optical absorption in synchrony with the heartbeat. Although the pulsatile absorption modulation is typically only a few percent of the baseline signal, it produces motions over scales that are relevant to fabrication. Brain pulsation alone displaces parenchyma by 1%–2% of tissue volume per cycle [55], while respiratory motion can exceed 1 cm in abdominal organs [55, 56]. Exposure to the laser itself adds further temporal variability. Inflammatory vasodilation begins within minutes of irradiation increasing local blood flow several‐fold and raising vascular permeability, while chronic optical window implants induce transient vasodilation (∼ 40% mean increase in vessel diameter) and edema in the first 2–3 days post‐implantation [57, 58].

The physiologically spatial scale relevant for photofabrication arising from the mechanical and thermal heterogeneities discussed in this section, is typically below 100 µm. This is set mainly by the diffusion length for oxygen and nutrients in vascularized tissue, typically below 100 µm from the nearest capillary [59]. Sampling coarser than 200 µm risks aliasing significant variations [60]. But the 100 µm capillary scale is only one level of a wider hierarchy which extends heterogeneity from sub‐cellular gradients at the voxel scale (∼ 1 µm), through capillary spacing (tens of µm), to organ‐scale architecture (∼ cm). Addressing this multiscale complexity requires not a single sensing modality but a hierarchical architecture integrating measurements across these scales [61, 62].

Despite the complexity of the interplay between the different axes, an architecture can be envisioned for the adaptive‐feedforward control. The thermal state can be directly and rapidly sensed and serves as the control quantity of the innermost control loop. The mechanical state provides the principal feedback. It spans nearly seven orders of magnitude across tissues, and it is not stationary, evolving as the host remodels.

It is accessible to online measurement (see §3.2.1). Refractometric and quantitative‐phase methods [63] track the refractive index rise that accompanies network formation in situ during fabrication, and vibrational spectroscopies resolve conversion [64] on millisecond timescales.

Refractive index responds to both conversion and temperature, so the phase signal on which such measurements rest is a superposition of chemical and thermal contributions and separating them requires an independent thermometric channel of the kind the architecture can already provide (see §3.2.2). The intravital measurements may require more specific correction strategies.

How can the initial values of writing parameters be obtained, if they cannot be presumed in advance? Before any energy is committed, the sensing modalities interrogate the tissue at doses below both the polymerization and the damage threshold, establishing the local optical, thermal, and mechanical baseline through perturbations that are reversible by construction (§5.1). Numerical predictions based on physical models can then be used to compute an initial exposure plan matched to the local environment, subsequently refined by the loop, like in object‐space dose optimization for volumetric additive manufacturing [65].

### 1.3 The Femtoliter Scale: Where Control Meets Biology

The femtoliter is not an arbitrary working unit but the natural scale at which the optics, biology, and engineering of intravital photofabrication intersect. A femtoliter (one quadrillionth of a liter, roughly one cubic micrometer) simultaneously matches the diffraction‐limited focal volume of a high‐*NA* two‐photon objective, the characteristic dimensions of cellular substructures, and the smallest spatial scale at which tissue thermal and mechanical properties remain continuous [21, 66, 67, 68]. The spatial scales of photopolymerization (∼1 µm) and tissue property variation (50–200 µm) differ by two orders of magnitude. This mismatch is not a limitation but an opportunity. It allows the sensing pathway to operate at lower numerical aperture than the writing beam, with relaxed objective and detector specifications, while still capturing the physiological heterogeneity at the relevant length scale.

Reaching the two‐photon polymerization threshold requires focal intensities of 10^11^ − 10^12^ W · cm^−2^ [69], which under realistic average powers can only be achieved with high numerical aperture (*NA* >0.8). As derived in **Box 2**, the two‐photon excitation rate scales as *NA* ^4^ while the focal volume scales as *NA* ^−4^, therefore the total radical generation per unit time is approximately *NA*‐independent. The accumulated radical density per voxel scales as *NA* ^3^ at fixed scan velocity and relaxing the focus below *NA* ≈ 0.8 therefore degrades polymerization efficiency rapidly while increasing voxel size and lowering fabrication resolution. Consequently, the sub‐micron voxel size in TPP is not merely a feature, it is a physical necessity imposed by the nonlinear optics.

The voxel geometry can also be deliberately engineered. Non‐diffracting beams [70, 71] like Bessel, Airy, and needle‐like profiles generated by axicons, spatial light modulators, or diffractive optics elongate the focal volume along the optical axis, producing high‐aspect‐ratio voxels (up to ∼ 500:1) in a single exposure [72]. The remaining challenge is not elongation itself but performing light delivery cleanly to avoid radial sidelobes, non‐uniform axial intensity, and low core efficiency that constrain resolution and exposure fidelity.

**BOX 2: Radical Generation and the *NA* Constraint**

**Radical Generation Rate**

The radical generation rate per unit volume scales quadratically with peak intensity and thus *NA* [4] with focusing optics. For pulsed excitation with duty cycle $d_{c}$:

$$G_{R}=\left(\Phi_{R}\sigma_{2}/d_{c}\right)\times {\left(\langle P\rangle \times \lambda /\mathrm{hc}\right)}^{2}\times NA^{4}\times \rho_{R}$$

where $\Phi_{R}$ is the radical quantum yield, σ_2_ the two‐photon cross‐section, and $\rho_{R}$ the photoinitiator density. Note that while the concentration rate scales as *NA* [4] the excitation volume scales as *NA* ^−^^4^. Therefore, the total number of radicals generated per unit time is approximately *NA*‐independent.

**Accumulated Radical Density**

During scanning at speed $v_{s}$, the residence time is *τ* $\approx \lambda /(\mathrm{NA}\cdot v_{s})$. The accumulated radical density per voxel becomes:

$$\rho_{1}=\left(\Phi_{R}\sigma_{2}/d_{c}\right)\times {\left(\langle P\rangle /\pi \mathrm{hc}\right)}^{2}\times \left(NA^{3}\times \rho_{R}\right)/\left(\lambda \times v_{s}\right)$$

For a representative high‐*NA* configuration (*NA*  =  1.2), a Gaussian beam (λ  =  800 *nm*) reaches a lateral waist of $w_{\mathrm{xy}}$ ≈ 155 nm and an axial extent $w_{z}$ ≈ 400 nm, given an effective two‐photon excitation volume $V_{eff}=\pi^{\frac{3}{2}}\;\cdot w_{xy}^{2}\cdot w_{z}\approx 0.05$ femtoliter (linear size of about 0.5 µm). The voxel volume increases to about 1 femtoliter when *NA* = 0.6 (linear size of about 1.3 µm) and 90 fL when *NA* = 0.2 (linear size of about 5 µm). This illustrates that femtoliter‐scale interaction volumes are an inherent consequence of the request of high spatial resolution in fabrication, rather than a limit required by optimization [73, 74].

The biological relevance becomes apparent when examining cellular organization. Mammalian cells typically occupy 30–4000 femtoliter (0.03‐4 picoliters) [75], with most adherent cell types clustering around ∼ 2000 femtoliter. Individual organelles fall squarely within the focal volume. Mitochondria occupy 0.2–2, endoplasmic reticulum encloses lumen 0.1‐1 fL, and primary cilia occupy 0.5‐2 fL. Photofabrication therefore acts at the level of single organelles, where local physical conditions directly govern cellular function.

Thermal physics at femtoliter scales exhibits unique characteristics that enable precise temperature control while minimizing collateral damage [35]. The thermal diffusion time across a 1 µm voxel (approximately 1 µs in tissue) is long compared with the 12.5 ns interval between laser pulses at typical repetition rates of tens of MHz, yet slow enough to resolve with current sensing technology. This creates a favorable control window: temperature perturbations can be detected and corrected within a single pulse interval.

The extreme thermal sensitivity of femtoliter volumes defines a narrow operational boundary (**Box 3**). The heat capacity of 1 fL aqueous solution (∼4 pJ · K^−1^) means that absorbing just ∼40 pJ raises temperature by 10°C which is the threshold for protein denaturation. A single 100 fs pulse carrying ∼1 nJ, depositing ∼10 pJ (≈1% local absorption), produces a 2–3°C transient rise [76]. At 80 MHz repetition rates and for pulse energies ∼100 pJ, even sub‐degree per‐pulse heating accumulates over ms‐scale dwell times to several kelvin above baseline, making active thermal management essential to limit biological damage. Small variations in local absorption coefficient or focal quality could even translate the optical setup from therapeutic polymerization 3D printing to destructive ablation. There is therefore a strong need for real‐time feedback rather than predetermined dosimetry.

**BOX 3: Femtoliter‐Scale Thermal Dynamics — Quantitative Framework**

**Heat Diffusion from a Confined Source**

For a spherical heat source of radius L, the characteristic diffusion time is:

$$\tau_{\mathrm{diff}}=L^{2}/6D$$

For tissue (*D* ≈ 0.14 mm^2^ s^−1^) and a 1 µm voxel (L = 1 µm):

$$\tau_{\mathrm{diff}}={\left(1\times 10^{-3}\text{mm}\right)}^{2}/\left(6\times 0.14{\text{mm}}^{2}s^{-1}\right)\approx 1.2\mu s$$

**Single‐Pulse Temperature Rise**

Assuming adiabatic heating (*τ*_pulse_ << *τ*_diff_), the temperature rise from absorbed energy *Q*:

$$\Delta T=Q/\left(\rho c_{p}V\right)=Q/C_{\mathrm{heat}}$$

**Table:** 

Parameter	Symbol	Value (1 fL)
Density × specific heat	*ρ c*p	4.2 MJ/(m^3^·K)
Volume	*V*	1 fL = 10^−^^1^ ^5^ L
Heat capacity	*C_heat_* = *ρ c*p *V*	**4.2 pJ/K**
Energy for *ΔT* = 10°C	*Q*_10_	**42 pJ**

**Example**: 1 nJ pulse, 1% local absorption → *Q* = 10 pJ → ***ΔT* ≈ 2.4°C**

**Cumulative Heating at High Repetition Rates**

Successive pulses overlap thermally because the inter‐pulse interval (∼12.5 ns at 80 MHz) is much shorter than the focal‐volume diffusion time *τ*_diff_ ≈ 1 *µs*. The cumulative temperature rise reaches a steady‐state value within microseconds of dwell, set by the balance between absorbed average power and diffusive heat removal, with experimentally validated heating coefficients of ∼15–18 mK per mW in mouse brain tissue, measured by both thermocouple and quantum‐dot thermometry under a 1‐mm^2^ scanned field [77] which is sufficient to require active thermal management at the µs timescale.

Single‐pulse adiabatic estimates underestimate the actual focal temperature: at 80 MHz repetition rates, the cumulative rise builds toward a steady‐state value within microseconds of dwell and crosses the protein‐denaturation threshold (10 °C above body temperature) within a few milliseconds at typical average powers of 10–50 mW.

**Thermal Confinement Condition**

For heating to be confined to the focal volume (minimal collateral damage), we need to have:

$$\tau_{\text{pulse}}\ll \tau_{\text{diff}}\ll \frac{1}{f_{\text{rep}}}$$

With *τ*_pulse_ ∼ 100 fs, *τ*_diff_ ∼ 1 µs, and 1/*f*_rep_ ∼ 12 ns (at 80 MHz), we satisfy the left hand but not the right hand of this inequality:

100fs≪1μs✓but1μs>12ns×

Failure of the right‐hand inequality at typical multiphoton repetition rates makes cumulative heating unavoidable and active thermal feedback essential.

**Sensing Requirements for Closed‐Loop Control**

**Table:** 

Requirement	Target	Rationale
Temporal resolution	**< 1 µs**	We are able to resolve *τ*_diff_
Temperature resolution	**< 1°C**	We can detect sub‐threshold increases of temperature
Spatial resolution	**< 10 µm**	We can localize the absorption within tissue compartments
Control bandwidth	**> 1 kHz**	Intervene before thermal damage accumulation (∼ 1–10 ms)

## 2 Failures in Photofabrication under the Open‐Loop Approach

### 2.1 Thermal Damage Cascades

Thermal damage in laser‐tissue interaction spans time scales from picoseconds (for non‐radiative decay) to minutes (for activation of inflammatory pathways), and length scales from nanometers (for chromophore oxidation) to millimeters (for the extension of necrotic zones) [77, 79]. In open‐loop fabrication, none of these intermediate stages is monitored and irreversible injury becomes detectable only after it has occurred.

**FIGURE 2:**
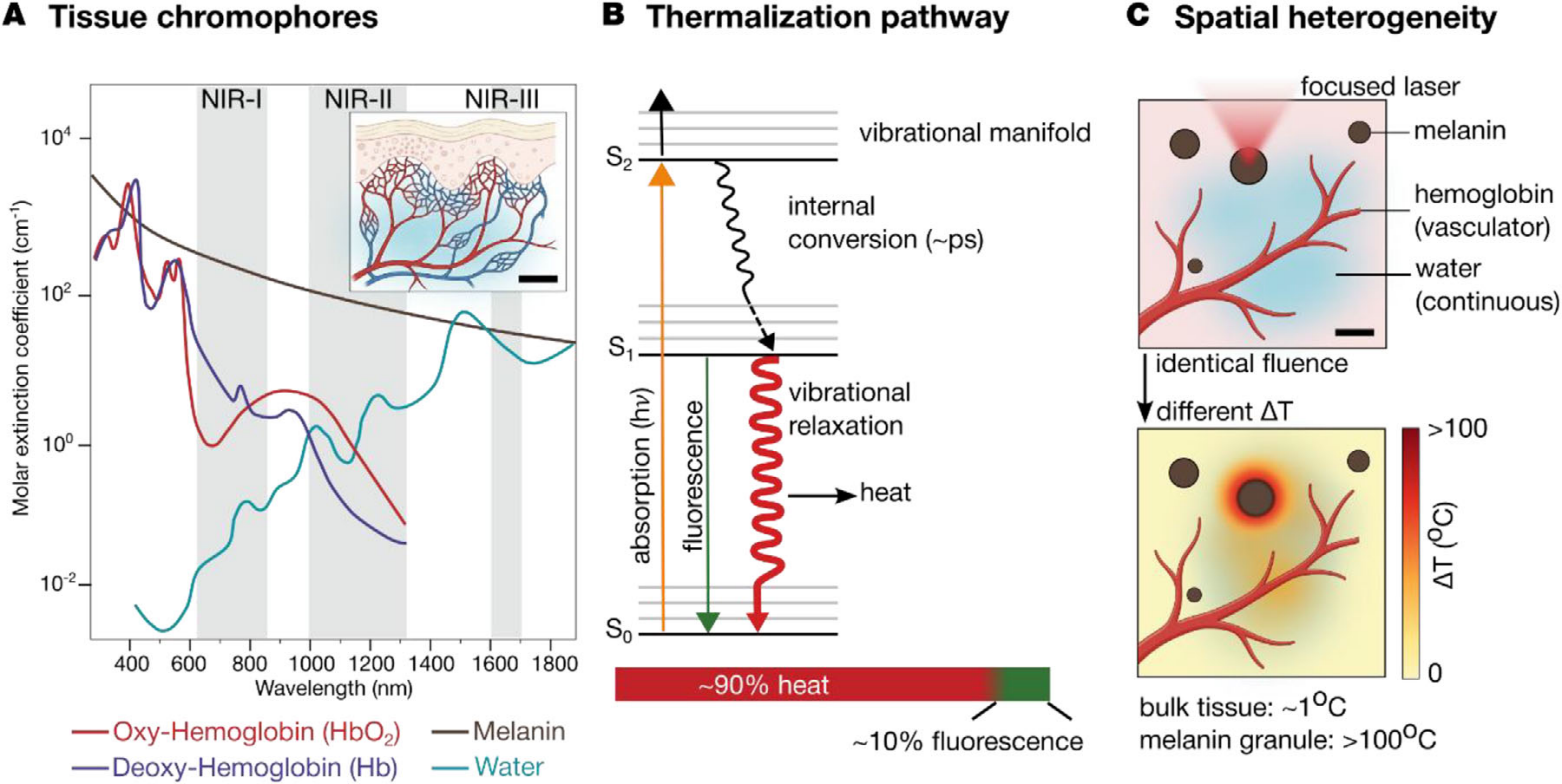
Photon absorption and thermalization in biological tissue. (A) Molar extinction coefficients of the dominant endogenous chromophores across the UV–vis–NIR window: oxy‐ and deoxy‐hemoglobin (HbO_2_, Hb) with characteristic Soret and Q‐band features and an isosbestic crossover near 800 nm; eumelanin, exhibiting a smooth broadband profile arising from structural disorder; and water, with characteristic OH vibrational overtone peaks in the NIR. Shaded bands mark the NIR‐I, NIR‐II, and NIR‐III tissue optical windows. Scale bar = 500 µm. (B) Modified Jablonski diagram of the post‐absorption pathway. Following electronic excitation (hν), ultrafast internal conversion within picoseconds funnels energy into vibrational modes, governed by the energy‐gap law *k*_nr_ ∝ exp(−*γ*·Δ*E*). Typical fluorescence yields near 0.1 imply that ∼90% of absorbed photon energy is dissipated as heat. (C) Consequence at the voxel scale: under identical incident fluence, discrete absorbers (e.g., melanin granules) experience temperature rises of hundreds of degrees, while the surrounding bulk tissue warms by only a few degrees, producing localized thermal hot spots and microcavitation invisible to bulk thermal models. Scale bar = 20 µm.

Energy deposition begins with photon absorption by endogenous chromophores like hemoglobin, melanin, and water which dominate in the near infrared (Figure 2A) [23, 32]. Once absorbed, the electronic excitation relaxes via ultrafast internal conversion. Energy funnels into vibrational modes within picoseconds, with non‐radiative decay rate following *k*_nr_∝exp (− *γ*Δ*E*) where *γ* is a molecular parameter related to vibrational modes and Δ*E* the energy gap between states. For most biological chromophores the fluorescence yield is of the order of 10%, so ∼ 90% of absorbed photon energy is converted to heat (Figure 2B,C). Thermal pathways therefore dominate even when photochemistry is the intended outcome of the irradiation.

The microscopic temperature rise, $\Delta \;T_{\mathrm{micro}}=\frac{\mu_{a}F}{\rho c_{p}}$, scales linearly with the light fluence *F* and linear absorption coefficient *µ*_a_ and inversely with the tissue density ρ and the specific heat coefficient *c*_p_. Under identical fluence (e.g., *F* ≈ 10 J · cm^−2^), the temperature increment spans three orders of magnitude across the spatial regimes encountered in intravital photofabrication. In tissues, largely composed of water (*µ*_a_ ≈ 0.1 − 0.3 cm^−1^, ρ ≈ 1 g cm^−3^,*c_p_* ≈ 3.2 J g^−1^ K^−1^) heating is modest (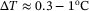) within the NIR transparency window (750 *nm* ≤ λ ≤ 850 *nm*) and readily cleared by perfusion. Photoinitiators effectively raise *µ*_a_ by two to three orders of magnitude, producing temperature increases of tens to over a hundred degrees, well into the regime of potential thermal damage. Discrete absorbers such as melanin granules with $\mu_{a}∼\;200-500\;cm^{-1}$ reach several hundred degrees, vaporizing intracellular water and nucleating micro‐cavitation bubbles that tear the surrounding structures away [80, 81, 82]. The same dose, the same physics, produces outcomes that span from imperceptible warming to mechanical disruption, depending entirely on the composition and the evolution of the tissue.

The biological consequence of sustained heating is described by the Arrhenius integral [83]:

(2) $$\Omega \;\left(t\right)=\underset{0}{\int^{t}}A\;\exp\left\{-\frac{E_{a}}{R}\;T(\tau )\right\}d\tau$$

In Equation (2), the parameter *A* is an empirically fitted frequency factor (typically 10^40^ − 10^100^ s^−1^ for proteins, reflecting the cooperative nature of unfolding rather than single molecule kinetics) and *E*_a_ is the activation energy for protein denaturation (200‐800 kJ · mol^−1^). The threshold Ω  =  1 corresponds approximately to the point at which ∼63% of the protein population is denatured. It marks the onset of irreversible damage [83]. The example of collagen is illustrative of the thermal sensitivity. With *E*_a_ = 630 kJ · mol^−1^  and *A*  =  1.2 × 10^98^ s^−1^, maintaining the fibrotic tissue at 65 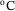 for just one second causes complete denaturation [84].

The cellular response to hyperthermia [85] involves multiple pathways. Mild hyperthermia (40
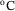
–42 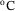) triggers the heat shock response through HSF1 trimerization and phosphorylation, inducing protective HSP expression [86]. Moderate hyperthermia (43
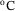
–45 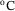) causes reversible injury through increased membrane fluidity, cytoskeletal disruption, and DNA repair failure. Severe hyperthermia (>50 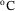) causes immediate coagulative necrosis through widespread protein denaturation and membrane destruction [87]. The vascular response amplifies and spreads damage. Initial vasodilation increases blood flow 2–5‐fold attempting to dissipate heat. Once vascular damage occurs (typically 48
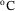
–50 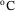), thrombosis and ischemia extend injury beyond the zone directly heated and a delayed inflammatory response extends tissue injury over hours to days after initial exposure [88].

Thermal denaturation is only one axis of damage. Photofabrication also drives a photochemical pathway in which the radical flux that sustains conversion, together with direct photo‐oxidative modification of biomolecules, damages cellular constituents by mechanisms that require no temperature rise and that accumulate with the global light and radical dose. Hyperthermia sensitizes cells to oxidative stress. A temperature and a photochemical dose that are individually tolerable may be detrimental when combined. In this case, the safe operating region is bounded by a curved surface in the joint space of temperature history and photochemical dose, rather than by a pair of independent thresholds. The precise shape of the safeguard boundary is not yet established for intravital conditions, and its characterization is a research problem in its own field.

### 2.2 Mechanical Mismatch and Delamination

Mechanical failure of intravital prints arises from the mismatch between the predominantly elastic‐plastic behavior of photopolymerized structures and the viscoelastic, poroelastic, and often anisotropic properties of living tissues. These disparities appear across multiple scales and evolve over timescales ranging from milliseconds during gelation, seconds to hours during shrinkage and full conversion and weeks to months during tissue remodeling and integration.

Polymerization produces volumetric shrinkage (**Box 4**) when van der Waals distances between monomers (3–4 Å) contract to covalent bond lengths (1.5 Å) [89]. The resulting volumetric strain $\frac{\Delta V}{V_{0}}$ scales approximately linearly with the degree of conversion (D, the fraction of the reactive monomers that have been transformed into polymer chains) and depends on monomer chemistry and functionality. For biocompatible resins reaching ≥ 70% conversion, $\frac{\Delta V}{V_{0}}$ typically falls in the 1–6% range, generating internal stresses in the ∼0.5–10 MPa range under constraint [90, 91]. The degree of conversion can be monitored through changes in refractive index, density, or vibrational signatures [92]. Among these, coherent Raman approaches are particularly suited for remote, label‐free monitoring [93] and have been demonstrated for laser fabricated microstructures in vitro [94].

The mechanical evolution of the network follows a well‐defined kinetic formulation. Gelation occurs at the Flory–Stockmayer threshold [95], typically at 20%–50% conversion for multifunctional monomers, depending on functionality and stoichiometry (**Box 4**). Beyond this point, the elastic modulus rises steeply as network connectivity grows. A common phenomenological form captures post‐gelation behavior:

(3) $$E\;\left(D\right)=E_{\infty }\;{\left(\frac{D-D_{gel}}{D_{final}-D_{gel}}\right)}^{m}$$

where $E_{\infty }$ is the limiting modulus, $D_{gel}$ and $D_{final}$ are the conversion fractions at gelation and at completion, and *m* ≈ 2.5 − 3 reflects the network topology (*m* ≈ 2.5 from percolation theory near gelation, *m* ≈ 3 from rubber elasticity at high conversion) [22, 96, 97]. The steep power‐law dependence (*m* >2) can be very useful in sensing: a 10% change in conversion near the gel point produces a 20%–30% change in modulus, making mechanical sensing a high‐sensitivity probe of polymerization completeness.

However, mechanical evolution at the tissue‐scaffold interface produces also stress amplification. Bi‐material interface mechanics predicts that for modulus ratios $\frac{E_{polymer}}{E_{tissue}}\geq 100$, the local stress intensity at the interface exceeds the far‐field shrinkage stress by more than an order of magnitude [98], with peak amplification at interface edge where debonding and microcrack initiation typically occur. Given that soft tissue tensile strength is 10–100 kPa and stiff photopolymers can generate MPa‐scale shrinkage stresses (**Box 4**), the modulus mismatch alone is sufficient to drive interfacial failure even in the absence of any thermal damage.

**BOX 4: Polymerization Mechanics — Quantitative Framework**

**Molecular Origin of Shrinkage**

Polymerization converts van der Waals contacts to covalent bonds:

$$d_{\mathrm{vdW}}\approx 3.4Å\to d_{\mathrm{covalent}}\approx 1.5Å$$

Linear contraction per bond: Δ*d*/*d* ≈ 56%. At the macromolecular network level, this local contraction is buffered by the non‐reactive monomer core and further diluted by crosslink density, unreacted functional groups, and solvent volume fractions. The resulting macroscopic volumetric shrinkage is described by:

$$\Delta V/V_{0}\approx D\varepsilon_{\max}$$

where D is the degree of conversion and *ε*_max_ the shrinkage at full conversion which is governed by the specific monomer chemistry [99, 100, 101].

**Table:** 

Material	ΔV/V_0_ (typical)	Governing factor
High‐water content hydrogels (e.g., GelMA, PEGDA)	1–6%	High water fraction dilutes polymer phase shrinkage
Methacrylate resins (e.g., Bis‐GMA, dimethacrylates)	2–9%	Bulky methyl group, steric hindrance, lower conversion
Acrylate resins (mono‐/multi‐functional)	8–15%	Higher reactivity and conversion, less steric hindrance

Consequently, acrylates shrink more than methacrylates at comparable functionality. Within each class, higher crosslink density and lower molecular weight between crosslinks increase shrinkage [102, 103].

**Flory–Stockmayer Gel Point**

The critical conversion $D_{gel}$ at which an infinite network forms:

$$D_{\text{gel}}=1/\left(f-1\right)$$

where *f* = monomer functionality (number of reactive groups).

**Table:** 

Functionality (f)	$D_{\mathrm{gel}}$	Example
*f* = 3	**50%**	Trifunctional acrylate
*f* = 4	**33%**	PEGDA, GelMA
*f* = 6	**20%**	Dipentaerythritol

**Shrinkage Stress in Constrained Geometry**

When adhered to tissue, volumetric shrinkage *ε* generates interfacial stress:

$$\sigma_{\mathrm{shrink}}\approx E\cdot \varepsilon /3\left(1-2\nu \right)$$

where *E* is the elastic modulus, *ν* the Poisson ratio, and the factor 3(1 − 2*ν*) reflects the bulk‐modulus relation for volumetric strain.

**Table:** 

Material/Tissue	Modulus	*σ*_shrink_
Soft hydrogels (GelMA)	1–10 kPa	0.1–1 kPa
Stiff hydrogels (PEGDA)	0.1–1 MPa	10–100 kPa
Rigid photopolymers	1–10 GPa	1–10 MPa
Soft tissue strength	—	10–100 kPa

### 2.3 The Reproducibility Crisis

The reproducibility crisis represents a fundamental barrier to clinical translation extending beyond simple technical variations to encompass biological variability, temporal dynamics, and complex nonlinear interactions. The success rate and the protocols’ reproducibility span very broad ranges: from near‐zero success rate in some contexts to as high as 80%–100% under favorable conditions [104, 105, 106, 107, 108, 109]. This variability underscores our limited ability to predict outcomes even for nominally identical procedures.

Biological variability arises across several scales. Genetic polymorphisms alter the abundance of key chromophores. Melanin content can vary by orders of magnitude between albino and pigmented strains, and heme‐binding protein levels vary depending on genotype and physiological state. Aging introduces additional heterogeneity through accumulation of advanced glycation end‐products (AGEs). Skin autofluorescence studies show AGE content increasing ∼10–15% per decade in healthy adults, with population reference values now established across the lifespan in cohorts exceeding 80,000 participants [110, 111]. In disease contexts, notably diabetes, AGE accumulation and extracellular matrix remodeling become more pronounced, increasing tissue heterogeneity and pathological risk.

Biological dynamics add time‐dependent variability on top of static heterogeneity. Circadian rhythms modulate core body temperature by 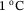 over 24 hours [112, 113, 114], affecting damage threshold. Peripheral blood flow varies by up to 40% across the circadian cycle [115, 116]. Hormonal cycles alter tissue hydration, vascular permeability, and inflammatory responses [117, 118]. Because many biological parameters interact multiplicatively, such as chromophore concentration, vascular density, scattering coefficient, metabolic state, even a moderate variability in any of the components (30%–50% coefficient of variation) produces much larger aggregated uncertainty. This compounding carries a direct statistical consequence. Treating each of *N* target parameters as Gaussian with standard deviation σ_*i*_ and an acceptance tolerance ± *δ*_*i*_, and assuming the parameters fail independently, the joint probability that all fall within tolerance is $P_{s}=\prod_{i}\mathrm{erf}\left(\delta_{i}/\sqrt{2}\sigma_{i}\right)$. Even when each parameter is individually met with 80–90% probability, *P*_s_ falls to 10–30% once *N* exceeds ∼10, a regime readily reached in intravital photofabrication where optical, thermal, mechanical, and biological tolerances must be satisfied at once. Positive correlation among failures, expected when the same physiological state drives several parameters together, only sharpens this decline. The predicted collapse is consistent with the low and variable success rates reported for complex preclinical and early‐clinical interventions [119, 120, 121]. The implication is that marginal improvement of any single parameter yields only limited payoff. Reproducibility instead demands adaptive closed‐loop control that compensates for the joint variability.

However, not all this variability needs to be tracked in real time. The variabilities governing the immediate outcome (temperature, modulus, geometry, local optical properties) are measurable during fabrication through the modalities of §3, while slowly varying biological factors such as chromophore abundance, glycation, and circadian phase enter instead as calibration priors fixed before fabrication begins (§5.1). This division of labor is what makes real‐time control tractable. The framework is nonetheless supported at present at the level of its components rather than as an integrated whole. The sensing and control primitives are individually demonstrated, but the integrated architecture has not been tested in a closed‐loop intravital setting, and we present it as a predictive hypothesis rather than a validated result (see §6).

## 3 Sensing Modalities: Reading the Femtoliter Voxel

Closed‐loop intravital photofabrication demands real‐time knowledge of the thermal and mechanical state within and around each printed voxel. In this section, we analyze sensing modalities that provide this information at the spatiotemporal scales relevant to femtosecond laser polymerization. We organize these modalities into two tiers: primary platforms that offer the integration potential, temporal response, and dual functionality required for practical closed‐loop control, and complementary modalities that extend sensing capabilities to regimes (deeper tissue, higher precision, passive monitoring) beyond the primary platforms' reach. This classification reflects both fundamental physical constraints and practical implementation considerations for systems operating on the microsecond‐to‐second timescales.

### 3.1 Design Principles for Femtoliter‐Scale Sensing

Before examining specific modalities, we establish four general principles that any sensing strategy must satisfy to implement closed‐loop control during photofabrication.

**Principle 1: Matched spatiotemporal sampling**. For multiple space and time resolved sensors, the Nyquist‐Shannon theory should be extended to a multidimensional parameter space [122]. For voxel dimensions of 300–800 nm laterally and 900–2000 nm axially, the Nyquist criterion demands spatial sampling at ≤ 200 nm to capture variations without aliasing [123]. Thermal diffusion across voxel dimensions occurs on microsecond timescales, requiring a sampling frequency of about 500 kHz. Practical implementations typically employ even higher (1–10 MHz) sampling rates [124]. Photon statistics considerations require spectroscopic rates of the order of 10 photons per microsecond. For NIR dyes we typically need excitation intensities of the order of 300 µW, at the upper end of the confocal microscopy excitation power range. Larger excitation powers will inevitably induce excessive photobleaching [32, 125].

**Principle 2: Non‐perturbative measurements**. The sensing process must not appreciably distort the target physical field. Taking thermometry as the representative example: for a continuous‐wave probe beam focused to a waist *w*_0_ ≈ 0.5 µm, steady‐state heat conduction limits the probe‐induced heating to 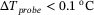 provided the probe power remains below ∼160 µW [126] under typical conditions (*µ*_a_ ≈ 1 − 10 cm^−1^, *k* ≈ 0.6 W m^−1^ K^−1^ for aqueous media). This bound tightens in highly absorbing regions (*µ*_a_ ≥ 100 cm^−1^), restricting power to a few microwatts. From the spectroscopic standpoint, this implies that the probe spectral window must be chosen carefully to reach sufficient photon statistics (see principle 1 and 3). The same non‐perturbation requirements apply to mechanical and optical probes, where excitation must remain below the level that alters the property being measured. This underscores the need for photon‐efficient approaches such as coherent detection or stimulated emission techniques [127, 128, 129].

**Principle 3: Information‐theoretic optimality**. A sensing strategy should extract maximum information about each parameter (temperature, Young's modulus, etc.) per detected photon, ultimately bounded by quantum efficiency and shot‐noise limit.

In absolute intensity‐based detection, the relative uncertainty of any parameter X is determined mainly by the shot‐noise [130, 131] and scales as $\frac{\delta X}{\left\langle X\right\rangle }\propto \frac{1}{\sqrt{N_{ph}}}$, where *N_ph_* is the number of photons. In terms of relative error on the spectroscopic measurement, the signal scales approximately with the average power at the probe volume 〈*P*〉, the spectroscopic cross‐section (*σ*) and the number concentration of probe molecules as $\approx \left(\frac{\left\langle P\right\rangle }{h\nu }\right)\;\sigma \cdot C\cdot w_{0}$. Assuming a MHz sampling rate that would allow time resolution of a few microseconds, a relative error ≃ 6% is obtained with *P* ≃300 µW, *σ*  = 10^−15^ cm^2^, and concentrations as low as *C* ≃ 10 *nM*. The actual resolution would then depend mainly on the efficiency of targeting the probe volume with the probe molecules (*C*), the excitation intensity reaching the probe volume <*P*> limited by absorption (affecting 〈*P*〉 directly) and by scattering (affecting indirectly the beam waist $w_{0}$) and the cross‐section *σ* (that depends on the excitation band).

Ratiometric schemes [132, 133] do not surpass this limit but approach it by rejecting source intensity fluctuations and drifts that otherwise dominate single‐channel intensity measurements. Phase‐sensitive detection offers a complementary route. Although ultimately shot‐noise limited, it rejects intensity noise and converts a small parameter change into a phase shift that can be amplified by optical path length. Phase is a continuous variable encoded in the field quadrature and can be estimated by uncertainty $\delta \varphi ∼\frac{1}{2\sqrt{N_{ph}}}$ and a thermo‐optic phase shifts $,\;\Delta \varphi =2\pi L\cdot \frac{\Delta T}{\lambda }\left(\frac{dn}{dT}\right)$ accumulates over the interaction length *L*, so that small temperature changes Δ*T* become resolvable at modest photon flux given a sufficiently long path. This underpins the superior temperature sensitivity of interferometric and OCT‐based approaches [134, 135, 136, 137, 138, 139].

**Principle 4: Multimodal complementarity**. No single sensing modality covers the entire spectrum of spatiotemporal scales, penetration depths, and sensitivities (**Box 5**). Optical methods ensure sub‐micrometer resolution but are confined to <1‐2 mm depth. Acoustic and photoacoustic techniques penetrate centimeters but sacrifice spatial resolution. Ultrafast modalities capture microsecond dynamics at the cost of signal‐to‐noise, whereas high‐sensitivity techniques demand long integration times. These irreducible trade‐offs necessitate hierarchical and complementary strategies that match each modality to the natural length and to the time scales it probes most efficiently [140, 141].

**BOX 5: Fundamental Sensing Trade‐offs**

**Table:** 

Modality	Resolution	Depth	Speed	Sensitivity
Optical (confocal)	<1 µm	<1‐2 mm	MHz	∼0.1‐1°C
OCT (phase)	∼10 µm	1‐3 mm	kHz‐MHz	<0.1°C
Photoacoustic	10‐100 µm	>9 mm	Hz‐kHz	∼1°C
NV diamond*	∼100 nm	Embedded	∼kHz	∼mK
UCNP	∼ 1µm	< 1 mm	∼ kHz	∼0.1‐1°C

*Legend*: Green = strength, Yellow = moderate, Red = limitation; * quantum Nanodiamonds Thermometry

**Key Physical Insight**

These trade‐offs are **largely irreducible** and arise from physics (diffraction limits, acoustic propagation, quantum measurement), not engineering limitations. Effective closed‐loop photofabrication in living tissue requires **hierarchical, multimodal sensing** that matches each modality to its optimal spatiotemporal regime.

Guided by these principles, we identify two primary sensing platforms: multimodal optical coherence tomography (OCT) and upconversion nanoparticles (UCNP) luminescence thermometry that together address the pulse train‐level (µs), voxel‐level (ms), and structure‐level (s) control loop defined later in §4. We then examine complementary modalities that extend capabilities to deeper tissue, higher precision, or passive quality monitoring.

### 3.2 Primary Sensing Platforms

The designation “primary” reflects three convergent criteria: (i) temporal response matching the microsecond‐to‐millisecond timescales of pulse‐ and voxel‐level control; (ii) compatibility with two‐photon polymerization optical paths, enabling co‐axial or dichroic integration without additional access ports; and (iii) demonstrated or within reach capability for real‐time feedback in intravital photofabrication contexts. Both platforms identified here, multimodal OCT and UCNP thermometry, satisfy these criteria while providing complementary information. OCT offers volumetric thermal and mechanical mapping from outside the material, whereas UCNP report temperature from within the polymerizing volume itself.

#### 3.2.1 Multimodal OCT: Unified Thermal and Mechanical Sensing

Optical coherence tomography provides a uniquely integrated platform for closed‐loop photofabrication: a single interferometric instrument can simultaneously extract thermal information (via temperature‐dependent phase changes) and mechanical information (via displacement tracking under applied or intrinsic stress). This dual functionality from one optical channel dramatically simplifies system integration compared to deploying separate thermal and mechanical sensors.

##### 3.2.1.1 Thermal Sensing via Thermo‐Optic Effect

OCT‐based thermal sensing exploits temperature‐dependent refractive index changes via the thermo‐optic effect, which is described by [142]:

(4) $$\frac{dn}{dT}={\left(\frac{\partial n}{\partial T}\right)}_{\rho }+{\left(\frac{\partial n}{\partial \rho }\right)}_{T}\cdot {\left(\frac{\partial \rho }{\partial T}\right)}_{P}$$

In Equation (4) the first term reflects the direct temperature dependence of electronic polarizability (typically negative) and the second (positive) arises from density reduction with temperature. In water and most biological tissues, these opposing contributions yield a net $\frac{dn}{dT}\approx -1.0\times 10^{-4}\;K^{-1}$ [143, 144].

**FIGURE 3:**
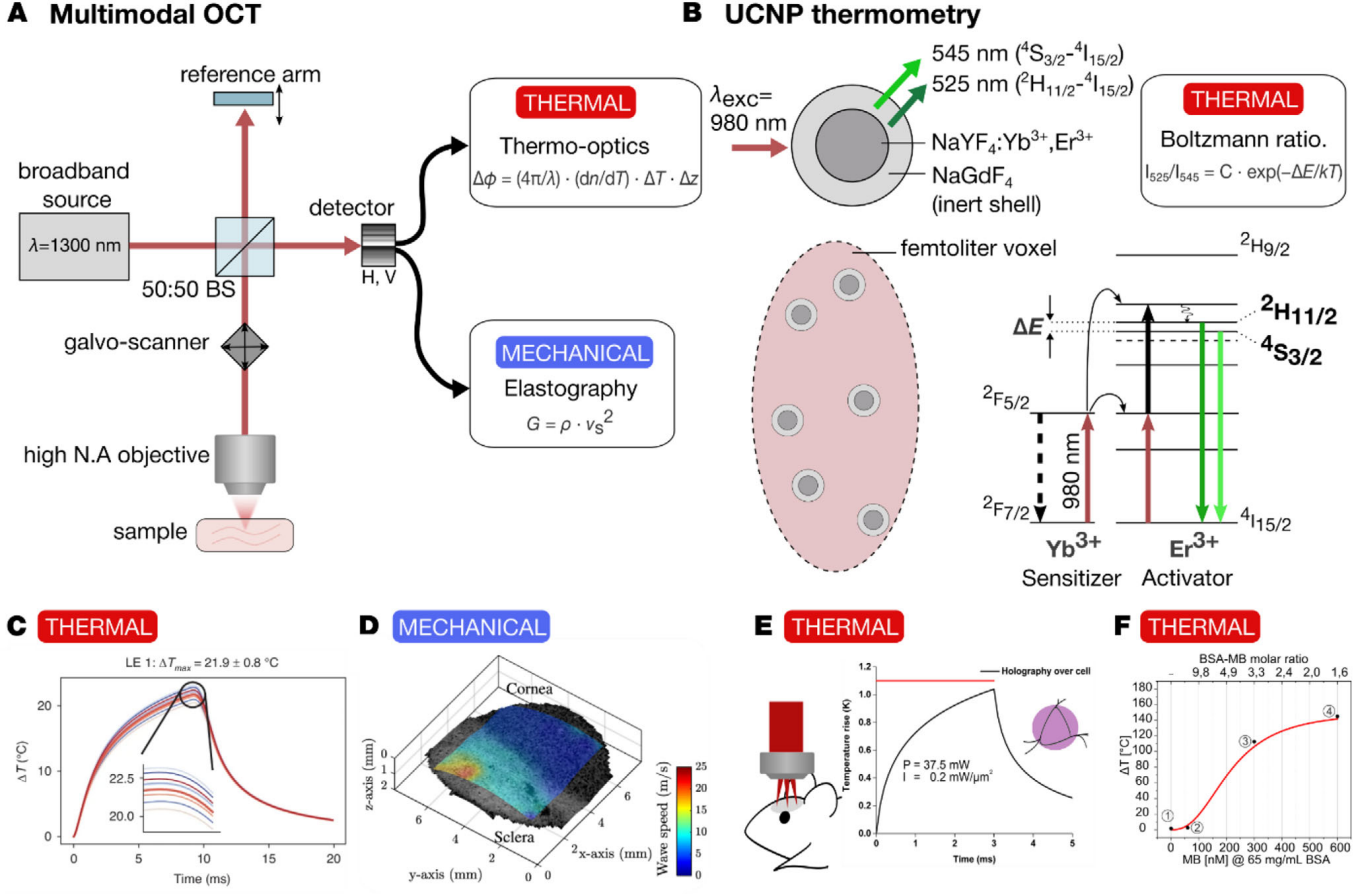
Primary sensing platforms for closed‐loop intravital photofabrication. (A) Multimodal optical coherence tomography extracts thermo‐optic phase shifts and phase‐sensitive elastographic strain from a single backscattered signal, providing volumetric thermal (Δ*φ* ∝ (d*n*/d*T*)·Δ*T*) and mechanical readout from outside the photopolymerizing volume. (B) Yb^3^^+^‐sensitized Er^3^^+^ upconversion nanoparticles act as embedded reporters: the Boltzmann‐distributed emission ratio R = *I*_5__2__5_/*I*_5__4__5_ from thermally coupled ^2^H_11__/__2_ and ^4^S_3__/__2_ levels provides self‐referenced thermometry immune to concentration and excitation fluctuations. (C) Sub‐degree retinal thermometry by phase‐sensitive OCT in vivo (Reproduced with permission [134], Copyright 2025, Springer Nature) and (D) air‐coupled ultrasonic OCT elastography for quantitative biomechanics (Reproduced with permission [149], Copyright 2020, Optica) (E) Nanothermometry in living organisms (Reproduced with permission [150], Copyright 2018, Cell‐press) and (F) direct in‐voxel temperature monitoring during multiphoton polymerization (Reproduced with permission [76], Copyright 2026, Wiley). Together, OCT and UCNPs provide complementary external/internal access to the thermo‐mechanical state of the printed volume.

Modern phase‐sensitive OCT exploits this thermo‐optic effect with interferometric schemes (Figure 3A,C) that preserve optical phase [134, 145]. Spectral‐domain and swept‐source implementations achieve phase stabilities approaching the shot‐noise limit of $\delta \varphi =\frac{1}{2\sqrt{N_{ph}}}$ rad, equivalent to $\frac{\lambda }{4\pi \sqrt{N_{ph}}}$ in optical path length. With typical near‐infrared systems (λ  =  1310 nm, 100 µW probe power, 10 µs integration), phase noise reaches ≈ 10 mrad ($N_{\mathrm{ph}}$ ≃ 2500), yielding path‐length precision below 2 nm and temperature resolution of ≈ 250 mK across 100 µm of tissue [146, 147]. Recent swept‑source OCT systems already exceed MHz scan rates, enabling real‐time volumetric imaging at near‑video rates [148].

##### 3.2.1.2 Mechanical Sensing via Phase‐Sensitive Elastography

The same phase‐sensitive detection that enables thermal measurements also ensures that elastography has an exceptional displacement sensitivity (Figure 3A,D) [151]. The phase change $\Delta \varphi =\frac{4\pi nd}{\lambda }$, where *n* is refractive index, *d* is axial displacement, λ the wavelength, yields ∼5.9nm displacement resolution, surpassing mechanical actuators [145, 152, 153]. Two complementary approaches can be employed. (i) Compression OCT‐elastography is based on loads applied through transparent windows and prediction of the stress distribution according to Hertzian contact mechanics, with deformation fields penetrating several contact radii into tissue. (ii) Dynamic OCT‐elastography induces propagating shear waves, whose velocity $v_{s}=\sqrt{G/\rho }$ enables real‐time stiffness mapping. For soft tissue (G=1−100kPa), propagation velocities of $1-10\;m\cdot s^{-1}$ allow millisecond‐scale mechanical characterization [154, 155].

##### 3.2.1.3 Integration With TPP System

The spectral separation between OCT wavelength (typically 1310 nm) and TPP writing beams (700‐900 nm) permits straightforward integration by simply inserting a dichroic into the optical path. A single objective can deliver both the printing and sensing beams: the OCT channel provides real‐time feedback on thermal gradients and mechanical evolution during laser polymerization. Recent innovations enable video‐rate mechanical imaging via parallel detection (line‐field/full‐field OCT), GPU‐accelerated phase unwrapping with <100 ms latency, and compressed sensing to reduce data requirements 10‐fold [156, 157, 158].

##### 3.2.1.4 Fundamental Limitations

Despite remarkable capabilities, OCT‐based sensing faces inherent constraints. Multiple scattering degrades coherence beyond 1–2 mm in tissue, restricting effective imaging depth and motivating alternative photoacoustic approaches at larger depths (§3.3.1). Speckle noise reduces phase precision in heterogenous structures, where microstructural variations can mask subtle thermal signals. Physiological motion including cardiac pulsation and respiration can corrupt measurements, necessitating sophisticated correction algorithms or gating strategies [159].

##### 3.2.1.5 Control Loop Mapping

OCT, with its kHz‐MHz acquisition rates and volumetric imaging capability, is best suited for voxel‐level (ms) and structure‐level (s) control loops. The ability to simultaneously monitor thermal gradients and mechanical integration provides the comprehensive state information required for adaptive control algorithms (described in §4).

#### 3.2.2 Upconversion Nanoparticle Thermometry

OCT senses temperature from outside the polymerizing volume through phase changes. Upconversion nanoparticles measure temperature directly from within the polymerized material itself. Embedded in photoresist or delivered to the target tissue, UCNPs serve as distributed molecular thermometers with inherent self‐calibration through ratiometric emission.

##### 3.2.2.1 Physical Basis: Boltzmann Thermometry

UCNPs exploit sequential absorption of multiple low‐energy photons through real intermediate electronic states yielding emission of higher‐energy photons. The hexagonal NaYF^4^: Yb^3 +^, Er^3 +^ system serves as a benchmark (Figure 3B). Here Yb^3 +^ absorbs at 980 nm and transfer energy to Er^3 +^, populating excited states that yield visible emissions including: green centered at 525 nm (from 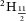) and 545 nm (from 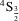), plus red emission centered at 655 nm (from 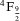) [160, 161]. Temperature sensing leverages thermal coupling between closely spaced energy levels (e.g., 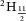 and 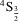 in Er^3 +^, separated by $∼800\;cm^{-1}$), governed by Boltzmann statistics:

(5) $$N_{H}/\;N_{S}=g_{H}\;/g_{S}\exp\left(-\Delta E/\left(k_{B}T\right)\right)$$

where *N_H_* and *N_S_* are the populations of the higher (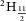) and lower (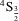) states, *g_H_* and *g_S_* their degeneracies, Δ*E* the energy separation, *k_B_* the Boltzmann constant, and *T* the absolute temperature. The intensity ratio $R=\frac{I_{525}}{I_{545}}=C\cdot \exp\left(-\frac{\Delta E}{k_{B}T}\right)$ directly yields temperature. The absolute sensitivity $S_{a}=\frac{d[\ln(R)]}{dT}=\frac{\Delta E}{k_{B}T^{2}}$ reaches $∼\;1.2\%\;K^{-1}$ at 300 K for Er^3 +^, among the highest for luminescent thermometers [161]. Crucially, the ratiometric approach provides inherent calibration against excitation power and particle concentration variations, a critical advantage for in vivo applications [150] (Figure 3E) where these parameters cannot be precisely controlled.

##### 3.2.2.2 Host‐Dopant Engineering for Optimized Performance

Beyond the benchmark NaYF^4^: Yb^3 +^, Er^3 +^ system, alternative combinations address specific limitations. Tm^3 +^ ‐doped systems enable NIR‐to‐NIR ratiometry for deeper tissue sensing where visible emission would be strongly absorbed. *Nd* ^3 +^ / *Yb* ^3 +^ pairs extend operation to higher temperature ranges. Core‐shell architectures (e.g., inert NaYF^4^ shells) suppress surface quenching, increasing quantum yields from ∼0.1% to >10% by isolating dopants from surface defects. Hexagonal‐phase hosts offer 10‐fold higher efficiency than cubic phases due to lower symmetry relaxing selection rules [161]. Dye‐sensitized UCNPs, leveraging organic dyes with absorption cross‐sections of 10^−^^1^ ^6^ cm^2^ (compared to 10^−20^ cm^2^ for Yb^3 +^), boost absorption 10,000‐fold and enable broadband excitation [162, 163].

##### 3.2.2.3 Biocompatibility and Integration Into Photoresists

UCNPs are exogenous compounds and integrating them into photoresists demands meticulous optimization of delivery, stability, and biocompatibility. PEG coatings (5–10 kDa) optimize circulation half‐life (∼4–24 hrs in vivo) while reducing opsonization and delaying immune clearance [164]. Targeting ligands, such as folate or RGD peptides, can direct nanoparticles to specific tissue niches, enhancing spatial selectivity. Colloidal stability, essential to prevent the aggregation that quenches upconversion emission, is achieved either by electrostatic repulsion [165] (charged ligands giving zeta potential |ζ| >30 mV) or by steric and hydration repulsion [166] (PEG and zwitterionic coatings, which stabilize at near‐zero net surface charge). Both routes should be verified by zeta‐potential and quantum‐yield characterization before integration. The integration of UCNPs into photoresists for real‐time closed‐loop thermometry during direct laser writing has been demonstrated [76, 167] (Figure 3F), establishing the practical foundation for the control architectures discussed in §4.

##### 3.2.2.4 Control Loop Mapping

UCNP ratiometric readout with millisecond temporal resolution can directly feed the voxel‐level control loop. With high‐speed detection systems, the response can approach the fastest control loop, though at the pulse‐train level UCNPs track the accumulated thermal envelope rather than individual pulses (see §4.1). The embedded nature of UCNPs, reporting temperature at the exact location of polymerization, provides information complementary to the label‐free, volumetric field reconstructed by OCT, which maps temperature across a region without requiring a probe at each point.

##### 3.2.2.5 Delivery, Spatial Uniformity, and in Situ Calibration

Even though in the ratiometric readout approach local concentration cancels out, density, and therefore controlled delivery, determines thermometric precision. Because the interband ratio is photon‐limited, the uncertainty of the temperature estimate scales inversely with the square root of the collected photon number, and sparse coverage broadens the estimate without displacing its mean. This concerns precision, the repeatability of the reading, not accuracy, its closeness to the true temperature. Whether the local particle density is sufficient should be verified directly from the achieved signal‐to‐noise ratio at each site: this depends on the tissue absorption and scattering and the thermal probe cross‐section in the specific spectral band (as discussed quantitatively in §3.1) a high probe density is therefore needed to meet the precision demanded by the innermost control feedback. Biodistribution reached via systemic administration is highly variable. The blood‐tissue barriers (e.g. thblood‐brain barrier) hinder altogether the diffusion of particles to specific tissues (brain, retina, testis etc.). Local injection achieves higher concentrations but establishes steep gradients. Image‐guided local deposition provides the placement control that the density criterion implies and represents the most realistic near‐term route [168].

Accuracy is governed by the calibration stability. Protein‐corona formation, photobleaching, and local variation in pH and refractive index all perturb the emission spectrum. Again, since temperature is read ratiometrically, perturbations that scale both bands equally (like concentration) cancel to first order. However, photobleaching is different for the two emitting states and prolonged interrogation can degrade them unequally. Corona formation and local variation in pH and refractive index act on the emission spectrum directly and are differential by nature. It is these residual differential effects that constitute genuine calibration drift. Such drift is detected during fabrication by cross‐comparison with the label‐free OCT thermal channel, which is independent of the luminescent readout and diverges from it as the calibration degrades. The disagreement provides a running estimate of the accumulated offset, which should be applied as a correction, while re‐establishing the calibration against a known thermal perturbation, as described in §5.1, requires a controlled reference and is performed between exposures.

These residual non‐idealities constitute the principal reason why the architecture cannot rely on upconversion thermometry alone. Temperature is measured redundantly, by upconversion luminescence and by label‐free OCT thermo‐optic phase, and the Bayesian fusion of §3.4 reduces the weight of the drifting channel accordingly.

### 3.3 Complementary Sensing Modalities

**FIGURE 4:**
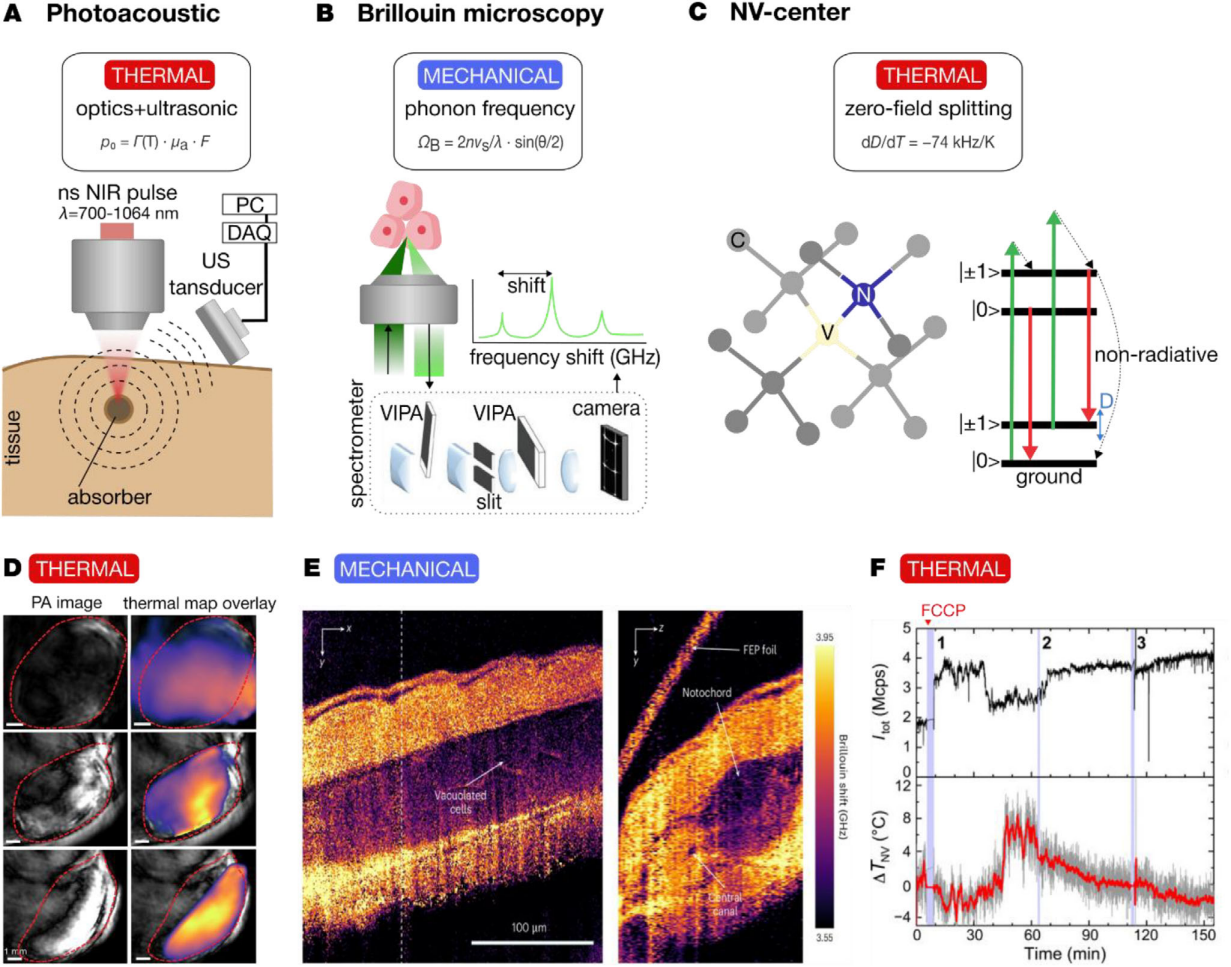
Complementary sensing modalities for closed‐loop intravital photofabrication. Three modalities extend sensing capability beyond the regime addressed by the primary platforms (OCT and UCNPs). (A) Photoacoustic imaging couples optical absorption to ultrasonic detection (*p*_0_ = *Γ*·*µ*_a_·*F*), providing thermal and mechanical readout at depths beyond the ∼2 mm reach of OCT through endogenous and exogenous contrast. (B) Brillouin microscopy detects inelastic scattering from thermally excited acoustic phonons (ν_B_ encodes longitudinal modulus *M*' and density *ρ*), offering simultaneous label‐free thermal–mechanical readout. (Reproduced with permission [169], Copyright 2019, Infocus) (C) Nitrogen‐vacancy (NV) diamond centers exploit the temperature‐dependent zero‐field splitting (d*D*/d*T* ≈ −74 kHz/K) read out by ODMR, delivering sub‐degree precision at single‐particle resolution. (D) Armored core–gold nanostar–mediated photoacoustic computed tomography enables whole‐body nanoparticle tracking, real‐time thermometry, and image‐guided photothermal therapy in deep tissue (Reproduced with permission [170], Copyright 2025, AAAS) (E) A Fourier‐transform imaging spectrometer achieves full‐field 2D Brillouin imaging at 40,000 spectra per second, ∼70 MHz precision, three orders of magnitude faster than confocal Brillouin microscopy (Reproduced with permission [171]. Copyright 2025, Springer Nature) (F) Nanodiamond quantum nanothermometers track temperature inside living C. elegans with ±0.22 °C precision and submicrometer localization (Reproduced with permission [172]. Copyright 2020, AAAS). Each modality fills a regime the primary platforms cannot including depth, dual‐readout, or ultimate precision.

The primary platforms, OCT and UCNPs, cover the core requirements for closed‐loop control but face inherent limitations in penetration depth, ultimate precision, and information channels. The following modalities extend sensing capabilities to regimes that complement rather than replace the primary platforms (Figure 4).

#### 3.3.1 Deep tissue access: Photoacoustic Thermometry

When optical penetration limits OCT to below ∼2 mm, photoacoustic (PA) thermometry extends thermal sensing to centimeter‐scale depths. The technique combines optical excitation with acoustic detection. Absorption of a nanosecond laser pulse induces thermoelastic expansion, launching acoustic waves detectable by ultrasound transducers (Figure 4A,D).

The initial pressure distribution *p*_0_ (*r*) =  *Γ*(*r*)*µ*_a_(*r*)*F* hinges on the local absorption coefficient *µ*_a_(*r*), the fluence *F*, and the Grüneisen parameter $\Gamma =\frac{\beta c^{2}}{c_{p}}$ (*β* is thermal expansion coefficient) [173, 174]. *Γ* is strongly temperature dependent and changes by a few percent per degree Celsius near body temperature [175] following:

(6) $$\frac{d\Gamma }{dT}=\Gamma \left(\frac{1}{\beta }\frac{d\beta }{dT}+\frac{2}{c}\frac{dc}{dT}-\frac{1}{c_{p}}\frac{dc_{p}}{dT}\right)$$

The scattering suffered by acoustic waves in tissues is two to three orders of magnitude weaker than that of optical photons, facilitating imaging at centimeter‐scale depths with resolution governed by acoustic rather than optical diffraction. The trade‐off between lateral resolution and depth of imaging can be approximately given by $\Delta x\approx \frac{0.5c}{f\cdot (NA)}$. Transducers working at *f* = 5–10 MHz with typical *NA* ≈ 0.3 − 0.5 yield 150–300 µm lateral resolution at 1–3 cm depth. Absolute PA thermometry already reaches nearly 0.6 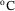 sensitivity at depths of centimeters in tissue [176]. State‐of‐the‐art PA systems deliver temperature maps at video rates, multispectral excitation disentangles thermal effects from chromophore variations, while machine learning extracts faint signals amid noise [170, 177, 178, 179, 180]. For closed‐loop photofabrication, photoacoustic monitoring serves the structure‐level control loop, providing deep‐tissue thermal surveillance when printing occurs beyond optical access, for instance through real‐time Kalman filter temperature estimation [181].

#### 3.3.2 Emerging Alternative: Brillouin Microscopy

Brillouin scattering provides simultaneous mechanical and thermal information through frequency shifts of photons inelastically scattered by phonons in the medium. The Brillouin frequency shift is given by:

(7) $$v_{B}=\frac{2nc}{\lambda }\;\sin\left(\frac{\theta }{2}\right)$$

where *n* is refractive index, *c* is sound velocity, λ is wavelength, and θ is the scattering angle. The dependence of *n* and *θ* on temperature enables thermal sensing with ∼ 0.1 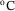 precision. Brillouin scattering also provides the longitudinal modulus that is related to the density and the (longitudinal) sound wave speed *M*  =  *ρ* *c* ^2^ (typically 2–10 GPa in hydrated tissues), without the need for external mechanical stress [182, 183]. Recent breakthroughs in stimulated and virtual‐imaging phased‐array (VIPA) configurations have reduced voxel acquisition times from seconds to milliseconds, enabling video‐rate 3D Brillouin mapping (Figure 4B,E) [184, 185]. Beyond advances in acquisition speed, the technique has been integrated with photoacoustic sensing [186] and combined with fluorescence and mass‐density mapping for correlative, cell‐type‐specific micromechanics [63].

An important caveat must be raised on Brillouin measurements that operate at GHz acoustic frequencies. The longitudinal modulus measured at these frequencies may differ substantially from quasi‐static Young's or shear moduli obtained by atomic force microscopy, rheometry, or tensile testing at Hz–kHz frequencies, since soft biomaterials exhibit frequency‐dependent viscoelastic behavior. Nonetheless, Brillouin microscopy provides valuable relative contrast and real‐time trend monitoring that are sufficient for closed‐loop feedback even without absolute calibration to low‐frequency mechanics.

#### 3.3.3 Quantum Frontier: Nanodiamond Thermometry

Nitrogen‐vacancy (NV) centers in nanodiamonds represent the ultimate‐precision frontier for thermal sensing, exploiting quantum spin physics to approach shot‐noise‐limited temperature resolution. The NV center forms a spin‐1 system with zero‐field splitting *D* ≈ 2.87 GHz between *m_s_* =  0 and *m_s_* =   ± 1 states. The splitting exhibits strong temperature dependence, $\frac{dD}{dT}\approx -74$ kHz·K^−1^, due to thermal expansion that alters the N‐V bond length and electron‐phonon coupling [172].

Thermometry by Optically Detected Magnetic Resonance (ODMR) achieves quantum‐limited sensitivity. Green laser excitation (532 nm) polarizes the spin into *m_s_* =  0 through spin‐selective intersystem crossing. Resonant microwave pulses then drive transitions to *m_s_* =   ± 1, and the spin state read out exploits spin‐dependent fluorescence with ∼ 5%–20% contrast (*m_s_* =  0 appears bright; *m_s_* =   ± 1 appears dark). The minimum detectable temperature change is

(8) $$\delta T_{min}\propto \frac{1}{\left|\frac{dD}{dT}\right|}\left(\frac{1}{C\sqrt{T_{2}^{*}t_{meas}}}\right)$$

where C is the ODMR contrast (∼ 0.05–0.2 for nanodiamonds), $T_{2}^{*}$ is the dephasing time (∼ 1 µs), and *t_meas_* is the total measurement time (with the square root reflecting shot‐noise scaling). One typically reaches sensitivities ∼ 5–50 mK/$\sqrt{\mathrm{Hz}}$ for single NV centers [187].

Several limitations confine nanodiamond thermometry to a complementary rather than primary role. The measurements require contact or near‐surface positioning due to microwave delivery requirements and limited optical penetration. The instrumentation complexity (pulsed lasers, microwave antennas, fast gating) exceeds that of optical‐only approaches. Nevertheless, biocompatibility has been extensively validated, cell viability exceeds 80% at concentrations up to 100 µ*g* · mL^−1^, with extraordinary chemical stability ensuring no degradation (Figure 4C,F) [172]. These properties position nanodiamonds as sensors for specialized applications demanding ultimate precision at accessible locations, and as a technology horizon for future closed‐loop systems.

Within the multi‐dimensional damage framework (§2.1), NV‐diamond relaxometry appears the natural sensor for the photochemical axis. It reports the local radical and oxidative‐stress load, and where an application requires that axis to be constrained explicitly, the channel is recruited alongside the primary thermal and mechanical platforms. The resulting primary‐complementary combination supplies the joint thermal and photochemical estimate against which the controller evaluates the safe operation surface.

#### 3.3.4 Passive Quality Monitoring: Acoustic Emission

Polymerization generates acoustic signatures through volumetric shrinkage and stress relaxation, information available passively without additional optical instrumentation. Unlike photoacoustic microscopy, which actively induces acoustic signals via pulsed laser absorption, acoustic emission (AE) monitoring captures the elastic waves intrinsically released as the network forms and internal stresses redistribute. In polymer and fiber‐reinforced systems, such emission spans the kHz‐MHz band and has long been used to detect microcracking, matrix failure, and delamination [188] (Figure 3D).

Machine‐learning classifiers trained on AE waveforms now distinguish distinct damage and failure modes with high accuracy [189], suggesting that an analogous approach could discriminate successful polymerization from thermal damage or incomplete conversion in real time [190, 191]. The inherently non‐perturbative nature of AE makes this monitoring modality readily integrable into existing bioprinting workflows as a parallel quality assurance channel complementing the optical primary sensors.

The actual implementation of these modalities into a real‐time loop is limited by the acquisition rate and by cross‐talk between channels. Regarding the acquisition rate, photoacoustic thermometry operates at Hz–kHz rates, appropriate to voxel‐ and structure‐level feedback. Brillouin microscopy, following the advent of VIPA and stimulated configurations, reaches millisecond voxel acquisition, suitable for the voxel loop and for the structure loop. Interfering optical channels can be disentangled by spectral deconvolution. When spectral separation is incomplete, time‐interleaving or lock‐in detection can be adopted. The non‐optical channels work on entirely distinct domains: photoacoustic transients and polymerization‐induced acoustic emission share the acoustic band and are distinguished instead by frequency content and timing (§3.3.4).

The pulse‐train level warrants a separate qualification. Thermometry based on up‐conversion nanoparticles and nanodiamonds both operate at kHz acquisition. Neither resolves individual pulse‐to‐pulse dynamics because sequential population of metastable states results in emission lifetimes of order 10^2^ − 10^3^µs. ODMR readout is similarly bounded. These channels track the cumulative thermal envelope from successive pulses, which evolves on a much slower timescale than laser pulses. At pulse‐train level the control is inherently limited to the reduced‐order thermal modes of Equation (9). The pulse‐train time scale is therefore the level at which the loop is least closed, and where the bandwidth gap between available sensing and the physics being controlled remains genuine.

### 3.4 Hierarchical Integration and Information Fusion

The primary‐complementary organization we have described maps directly onto a hierarchical sensing architecture spanning from quantum to macroscopic scales, each tier governed by distinct physics and matched to specific control loop requirements (**Box 6**). At nanoscale (1–100 nm), quantum sensors provide ultimate precision. Single NV centers in 5 nm nanodiamonds achieve below $1\;\frac{mK}{\sqrt{Hz}}$ through quantum coherent protocols [192] which is a complementary capability reserved for applications demanding the highest precision at accessible locations. At microscale (0.1–10 µm), the primary UCNP platform provides distributed sensing networks at concentrations of 10^6^ − 10^8^ particles · mm^−3^ it reaches cellular‐resolution temperature mapping with millisecond response. Each UCNP particle (20–50 nm size) reports local temperature through ratiometric emission [161, 193], insensitive to concentration or excitation variations. This makes UCNPs the workhorse of thermal sensing for pulse‐ and voxel‐level control. At mesoscale (10–1000 µm), the primary OCT platform provides three‐dimensional thermal and mechanical mapping through phase‐sensitive detection with sub‐degree resolution [134] and 5–20 µm [194, 195, 196] spatial resolution. Brillouin microscopy offers a complementary emerging alternative with dual thermal‐mechanical readout from a single optical channel. At macroscale (>1 mm), photoacoustic thermometry extends sensing to depths beyond optical penetration limits (>10 mm with 150–300 µm resolution) [197], serving structure‐level monitoring when deep tissue access is required.

#### 3.4.1 Sensor Fusion Framework

Combining information from multiple sensors requires algorithms that account for their disparate uncertainties, sampling rates, and noise characteristics. A Bayesian framework handles this naturally. The posterior over the tissue state *X* given all measurements *Z* follows P(X|Z)∝P(Z|X)P(X), propagating heterogeneous measurement uncertainty into a single state estimate [198, 199]. Assuming conditional independence between sensors, the likelihood factorizes as $P\;\left(Z|X\right)=\;\prod P\left(Z_{i}|h_{i}\left(X\right)\right)$ where the observation model $h_{i}$ maps complete tissue state to quantities measured by sensor i. Where these observation models are nonlinear or only partially known, deep neural networks [200] can learn them directly from data, capturing inter‐sensor dependencies not easily expressed in closed form, at the cost of requiring training data and reduced interpretability.

Implementing such fusion in real time requires matching the computation speed to the nested control timescales of the system. The fast loop (pulse train‐level thermal management at sub‐microsecond latency) can map onto field‐programmable gate array (FPGA) which provide deterministic, low latency processing of high‐bandwidth sensor streams [201]. Voxel‐ and structure‐level image reconstruction, by contrast, is massively parallel and maps onto graphics processing units (GPUs). High‐bandwidth interconnects and ring‐buffered data pipelines decouple acquisition from processing so that no single stage stalls the loop [202, 203]. This heterogeneous computing architecture mirrors the timescale hierarchy of the control problem itself: deterministic hardware for the fast loop, parallel hardware for the slower reconstruction layers.

Integration into a single platform is more tractable than a modality‐by‐modality reading suggests, because the optical channels share one excitation‐and‐collection path. OCT operates near 1310 nm and the fabrication beam at 700–900 nm, therefore a single dichroic and a single objective already deliver both (§3.2.1). Brillouin scattering and NV fluorescence are excited through that same objective using additional laser lines, and the returning signals travel back along the shared path to be separated downstream by a dichroic beamsplitter, each routed to its own detector or spectrometer, as is standard in multimodal microscopy. The non‐optical sensing platforms are entirely decoupled from the optical path hardware. Photoacoustic detection requires an acoustic transducer and NV ODMR a microwave antenna, neither of which perturbs the optical beam path. The realistic short‐term configuration is the integrated OCT‐and‐UCNP primary core extended by photoacoustic detection for deep targets.

The possibility of this integration is already partially established. Multispectral photoacoustic microscopy and OCT driven by a single megahertz supercontinuum laser through one shared path, the 500–600 nm band serving photoacoustic excitation and the 600–840 nm band serving OCT, has been demonstrated in vivo in mouse ear [204]. The same architecture has been reduced to a ∼2 mm probe in which OCT and optical‐resolution photoacoustic microscopy share a common optical path while photoacoustic detection is performed by a transducer outside it [205]. Intervention has been integrated on similar bases: a 532 nm therapy laser and OCT delivered through a single double‐clad fiber, therapy light in the inner cladding and OCT in the core, achieve inherent co‐registration and identify coagulation depth in real time, terminating exposure automatically when the target depth is reached [206].

**BOX 6: Hierarchical Sensing Architecture**

Closed‐loop photofabrication requires sensing across multiple spatial scales, each governed by distinct physics. No single modality spans all scales, effective control demands hierarchical integration:

**Table:** 

Modality	Tier	Scale	Control loop	Information
**Multimodal OCT**	Primary	Meso (10‐1000 µm)	Voxel + Structure	Thermal + Mechanical
**UCNP**	Primary	Micro (0.1‐10 µm)	Pulse + Structure	Thermal (embedded)
**Photoacoustic**	Complementary	Macro (>1 mm)	Structure	Thermal (deep)
**Brillouin**	Complementary	Meso (10‐1000 µm)	Voxel	Thermal + Mechanical
**NV diamonds**	Complementary	Nano (1‐100 nm)	Pulse + Voxel	Thermal (precision)
**Acoustic Emission**	Complementary	N/A (passive)	Quality monitoring	Polymerization state

**Key Physical Insight**

Each scale is governed by **distinct physics**: near‐field thermodynamics (nano), statistical mechanics (micro), continuum optics (meso), wave propagation (macro). Effective closed‐loop control requires **matching sensor physics to target phenomena**, then fusing information through probabilistic frameworks that propagate uncertainties across scales.

## 4 Control Architectures: From Sensing to Adaptation

We move now to discuss what is the extent and the accuracy with which we can monitor the temperature and mechanical strain on the polymerized material “on the fly”.

### 4.1 The Closed‐Loop Control Paradigm

**FIGURE 5:**
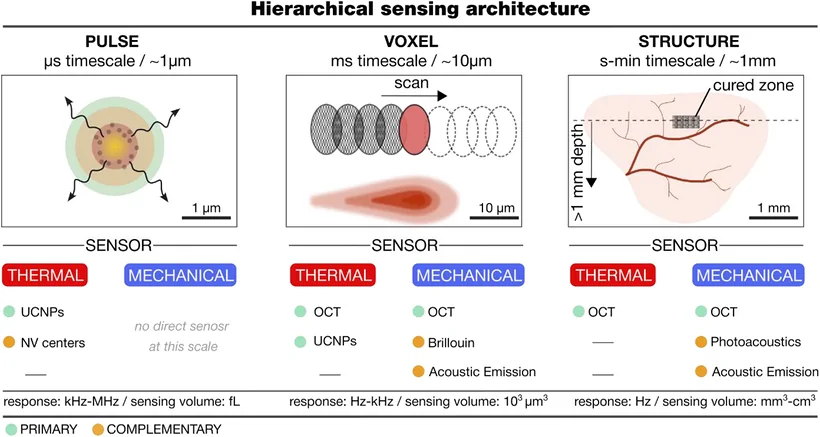
Hierarchical sensing architecture for closed‐loop intravital photofabrication. The three control loops operate at distinct timescales: pulse‐level management (µs), voxel‐level feedback (ms), and structure‐level validation (s–min), ordered from the fastest layer to the slowest (left to right). Thermal sensing modalities are shown (below, on the left), mechanical sensing modalities (below, on the right). PRIMARY platforms (green) denote integration‐ready technologies suitable for practical closed‐loop implementation: upconversion nanoparticles (UCNPs) for embedded ratiometric thermometry, and multimodal optical coherence tomography (OCT) for simultaneous thermo‐optic phase detection and elastographic strain mapping. COMPLEMENTARY modalities (yellow) extend capability to specialized regimes including nitrogen‐vacancy (NV) diamond centers for millikelvin precision, Brillouin microscopy for label‐free dual mechanical readout, and acoustic emission (AE) for passive polymerization monitoring, while photoacoustic (PA) imaging provides the complementary pathway for deep tissue (∼1 cm) where optical access is limited. At the pulse‐train level, the luminescent channels (UCNPs and NV centers) report the cumulative thermal envelope across successive pulses rather than resolving individual pulse‐to‐pulse dynamics.

Closed‐loop intravital photofabrication replaces the open‐loop sequence (plan → print → assess) with a continuous feedback architecture in which sensor measurements continually adapt laser actuation. The natural architecture is hierarchical. The dynamics of the system span more than seven orders of magnitude from sub‐microsecond for per‐pulse heating, through millisecond for polymerization kinetics, to seconds‐to‐minutes for the mechanical equilibration with surrounding tissue. Such timescale separation is the canonical condition under which hierarchical control admits a decoupled design [207, 208], with each loop operating on a single dominant timescale while the slower loop provides setpoints to the faster loops. We therefore organize sensing and actuation into three hierarchical control loops, ordered by characteristic timescale (Figure 5).

We use here loop control as a limit of what could be more consistently defined as adaptive feedforward with supervisory feedback. The measured quantities are not independent proxies for a hidden outcome but coupled observables of one process, radical polymerization, which raises the elastic modulus through the constitutive relation of Equation (3) and simultaneously releases reaction heat. Therefore, the same dose that advances conversion also deposits thermal energy. Three quantities can then be distinguished by role. Temperature is a process condition, coupled to the conversion rate through the reaction exotherm. The mechanical modulus and the geometry are direct fabrication outcomes, measured online by OCT‐elastography and Brillouin microscopy (§3.2.1, §3.3.2), and for intravital tissue fabrication the mechanical result is frequently the property that determines success (§2.2). The loop closes upon it directly, though, in terms of radical cross‐linking, it is better regarded as adaptive feedforward. The architecture therefore closes genuinely on the mechanical outcome and reconstructs the chemical one, rather than substituting temperature for either.

#### 4.1.1 Pulse‐Train Loop (µs)

The fastest loop regulates the cumulative thermal load produced by successive laser pulses, modulating per‐pulse energy, repetition rate, or burst gating to avoid thermal damage of the tissue within the focal volume. UCNP ratiometric thermometry is the primary sensor at this timescale. The embedded probe reports temperature from within the polymerizing voxel itself, with kHz‐scale acquisition approaching the loop bandwidth [209, 210]. NV‐diamond thermometry provides a complementary high‐precision channel (∼30mK at 1 kHz) where optical and microwave access permit ODMR readout [211, 212]. The model exploited by this inner loop is a reduced‐order representation of the Pennes bioheat dynamics, a proper orthogonal decomposition (POD) [213, 214]:

(9) $$T\;\left(r,t\right)=\bar{T}\;\left(r\right)+\sum a_{i}\left(t\right)\varphi_{i}\left(r\right)$$

where φ_*i*_(*r*) are spatial modes learned from simulations and *a_i_* (*t*) denote time‐varying coefficients estimated from sensor data. Equation (9) enables real‐time model predictive control (MPC), formulated explicitly in §4.2.

#### 4.1.2 Voxel Loop (ms)

The middle loop evaluates the degree of polymerization and the mechanical state of each voxel, adapting dwell time, scan velocity, or local exposure. Multimodal OCT is the primary platform where a single backscattered signal yields simultaneous thermo‐optic phase ($\Delta \varphi \propto (\frac{dn}{dT})\cdot \Delta T)$ and elastographic strain at acquisition rates of 100 kHz‐1 MHz with current swept‐source instrumentation [147, 215], a combination unique among optical modalities. Brillouin microscopy and acoustic emission provide complementary readouts when passive process integrity is required.

#### 4.1.3 Structure Loop (s‐min)

The slowest loop evaluates 3D geometry against the target design and adjusts the scan trajectory or, if necessary, aborts the procedure. OCT volumetric imaging is the primary platform in which full‐field and line‐field implementations enable video‐rate 3D mapping. For deep intravital applications beyond the ∼ 2 mm penetration of OCT, we should resort to photoacoustic tomography.

The hierarchical setpoint flow runs from slower to faster loops (slower loops set the targets for faster ones), while information flows from faster to slower loops (faster loop report their state upward). §4.2 details the algorithms executed within each loop.

### 4.2 Control Algorithms

The three constituent loops introduced in §4.1 require corresponding control algorithms. Model predictive control and adaptive dual control paradigms are mature enough for near‐term deployment in intravital photofabrication. As an alternative, learning‐based control via physics‐informed neural networks (PINNs) [216], is emerging but not yet ready for safety‐critical use.

#### 4.2.1 Model Predictive Control (MPC)

MPC anticipates the thermal response of the focal volume over a prediction horizon of *N* = 10–100 future steps, selecting the input sequence that minimizes tracking error while respecting safety constraints [217]. MPC has been deployed for real‐time thermal regulation in laser‐based additive manufacturing [218, 219]. In simple terms, MPC operates like a driver watching the road ahead rather than the rear‐view mirror. At each instant it uses a model to infer how the focal‐volume temperature will evolve, looks several steps into the future and select the laser‐power sequence that keeps the temperature within the required (safety) boundaries. Only the first step of that sequence is applied before the prediction is repeated, and the controller continuously re‐plans as new sensor data are acquired. Let $\hat{T}\left(k+i|k\right)$ denote the model‐predicted temperature at future step k+i, conditioned on measurements available at the current step k, and let P(k+i) denote the laser power applied at that step. At each control cycle k, MPC solves the following finite‐horizon optimization:

(10) $$\begin{array}{c} \mathrm{mi}n_{P\left(k+i\right)}\left(\sum_{i=1}^{N}∥\hat{T}\left(k+i|k\right)-T_{\mathrm{ref}}\left(k+i\right)∥\begin{array}{c} 2\\ Q \end{array}+\sum_{i=0}^{N-1}∥\Delta P\left(k+i\right)∥\begin{array}{c} 2\\ R_{u} \end{array}\right) \end{array}$$

In words, the first term penalizes deviation of the predicted temperature from its target, and the second penalizes abrupt changes in laser power, enforcing smooth and physically realizable actuation. Here, ${∥\cdot ∥}_{Q}^{2}$ and ${∥\cdot ∥}_{R_{u}}^{2}$ denote weighted quadratic norms (${∥x∥}_{Q}^{2}\equiv x^{⊤}Q_{x}$) subject to the predicted dynamics:

(11) $$\hat{T}\;\left(k+i+1|k\right)=M\left(\hat{T}\left(k+i|k\right),\;P\left(k+i\right),\;\xi \right)$$

and to safety constraints:

(12) $$T_{min}\leq \hat{T}\left(k+i|k\right)\leq T_{max},\;P_{min}\leq P\left(k+i\right)\leq P_{max}$$

These inequalities encode the hard safety limits. The predicted temperature and the applied power must remain within bounds at every step. This is precisely the property that distinguishes MPC from simpler controllers. The positive‐definite weighting matrices *Q* >0 and R_*u*_ >0 set the relative penalties on, respectively, deviations from the target temperature trajectory $T_{\mathrm{ref}}$ and fluctuations in the applied power ΔP(k+i)=P(k+i)−P(k+i−1) to enforce smooth actuation. M denotes the reduced‐order thermal model introduced in §4.1, Equation (9), parametrized by tissue photothermal coefficient vector *ξ*. This vector collects the tissue properties that govern the local thermal response to laser exposure, principally the optical absorption coefficient, the thermal conductivity, and the blood‐perfusion rate grouped together because they enter the bioheat model (Equation (1)) as the unknowns that map a given laser power to a resulting temperature field. Only the first computed control input P(k) is applied before the optimization is repeated at the next step (receding horizon principle), and the measured temperature $T_{\text{measured}}\left(k\right)$ is fed back to initialize the prediction $\left(\hat{T}\;\left(k|k\right)=T_{measured}\;\left(k\right)\right)$ at each cycle. Hard limits are encoded as inequality constraints inside Equation (12) of the MPC formulation, bounding the photochemical as well as the thermal component of the damage state (§2.1), and are enforced at the hardware level by independent inspectors that gate the laser driver outside the software control path.

Robust variants extend Equation (10) to stochastic or tube‐based formulations to account for sensor noise and model error [220]. GPU‐parallelized quadratic programming solvers such as OSQP [221] and qpOASES [222] evaluate Equation (10) in microseconds, enabling real‐time deployment [219, 223, 224, 225]. MPC alone assumes that the photothermal coefficient *ξ* is known a priori. Unlike the controllable input, *ξ* is set by the tissue itself. It varies between patients and between sites, and drifts during a procedure as perfusion and damage evolve. This assumption therefore fails in vivo, which motivates the adaptive layer (§4.2.2) to estimate *ξ* in real time.

#### 4.2.2 Adaptive Dual Control

The term “dual” refers to a controller that pursues two goals at once. It acts to regulate the process while simultaneously probing the system to learn the tissue properties it does not yet know. Adaptive dual control [226] fuses real‐time parameter estimation with process regulation, enabling the system to learn patient‐ or bioink‐specific properties on the fly. The algorithm evolves by sequentially updating the estimated state of the focal volume, $\hat{x}\left(k\right)$ (the local thermal and, where available, mechanical condition), the tissue or microstructure features, $\hat{\xi }\left(k\right)$, and the writing parameters, *u*(*k*). At each step *k*, two coupled updates run in parallel:

(13) $$\hat{\xi }\left(k+1\right)=\hat{\xi }\;\left(k\right)+K\left(k\right)\left[y\left(k\right)-\hat{y}\left(\hat{\xi }\left(k\right)\right)\right]$$

(14) $$u\;\left(k\right)=f\left(\hat{x}\left(k\right),\hat{\xi }\left(k\right)\right)+u_{probing}\left(k\right)$$

In plain terms, Equation (13) is how the controller updates its best estimate of the tissue properties. It compares what the sensor measured against what its current model predicts and nudges the estimate to close that gap. Equation (13) is a recursive parameter update. The estimate $\hat{\xi }\left(k\right)$ is corrected by the residual between the measured sensor output y(k) and its model prediction $\hat{y}\left(\hat{\xi }\left(k\right)\right)$, weighted by the observer gain K(k), typically derived from extended Kalman filtering [227] or recursive least squares [228]. Here y(k) is the directly measured quantity (for example the UCNP emission ratio or OCT phase), related to the underlying state $\hat{x}\left(k\right)$ through the sensor observation model. The state itself is not observed directly but reconstructed from these measurements. Equation (14) is the dual control law. The control input u(k) (i.e. laser power, scan velocity, or fabrication position) combines the MPC‐optimal action $f(\hat{x}\left(k\right),\hat{\xi }\left(k\right))$, computed from the current state and parameter estimates, with a small dither signal $u_{\mathrm{prob}\mathrm{ing}}\left(k\right)$ (pseudo‐random, sinusoidal, or chirped) that maintains persistent excitation and guarantees identifiability of $\hat{\xi }\left(k\right)$ in steady state [229]. Within milliseconds to seconds, the initial nominal model is replaced by a continuously refined, specimen‐specific description, reducing the reproducibility gap that characterizes open‐loop fabrication.

#### 4.2.3 Learning‐Based Control

As a third emerging paradigm, physics‐informed neural networks can be trained offline to serve as differentiable surrogates for the Pennes bioheat equation. They embed the governing PDE directly into the loss function rather than learning from data alone [230]. Recent applications to laser‐tissue interaction include parametrized 3D models of laser‐irradiated skin with embedded vasculature and tumors [231] and PINN‐based reconstruction of internal temperature fields from sparse surface measurements [232]. In laser‐based additive manufacturing, a neural‐network surrogate (TiDE) has been integrated into a multi‐step MPC framework for melt‐pool temperature control with porosity‐defect mitigation, providing the closest demonstrated analogue to a closed‐loop intravital digital twin [233]. For intravital photofabrication, the absence of validated training datasets and the safety‐critical nature of the application currently restrict learning‐based control to offline simulation environments. As validated datasets accumulate, hybrid architectures that combine a model‐based core (MPC + adaptive) with learning‐based correction layers represent the most plausible long‐term direction [234].

### 4.3 Safety Layers and Failure Management

A safety‐critical control architecture for intravital photofabrication can be structured in four operating tiers. (i) Hard limits are encoded as inequality constraints inside Equation (12) of MPC formulation and are enforced at the hardware level by independent inspectors that gate the laser driver outside the software control path. (ii) Soft constraints are realized through tube‐based or chance‐constrained MPC variants that maintain a guaranteed safety margin as parameters approach the hard limits [235, 236], and through Control Barrier Functions that act as terminal safety filters on the actuator command [237]. (iii) Anomaly detection monitors the multimodal sensor stream for deviations from the learned nominal signature. Deep representation learning has demonstrated >95% real‐time detection accuracy on analogous laser‐based manufacturing processes [238, 239]. (iv) Graceful degradation completes critical structural elements while suspending non‐essential refinements when sensor confidence drops, an approach drawn directly from safety‐critical surgical robotics [237].

## 5 Implementation Strategies

The control and safety architecture of §4 must be embedded within a clinically realistic workflow that starts hours before fabrication and may last weeks for post‐operative follow‐up. The closed‐loop architecture maps onto three temporal phases: pre‐printing sensor deployment and in situ calibration (§5.1), intra‐operative real‐time control (§5.2), and post‐printing longitudinal validation (§5.3). §5.4 surveys near‐term and emerging applications.

### 5.1 Pre‐Printing Preparation

Closed‐loop control begins 24–48 hours before procedures with deployment of optical thermometers and chemical reporters into the target tissue. For systemic administration, surface‐functionalized UCNPs (5–15 mg · kg^−1^) are infused intravenously over 30–60 min. PEG coating (5–10 kDa) extends the circulation half‐life to several hours, and zwitterionic phospholipid coatings further extend residence time while reducing opsonization and macrophage clearance [240, 241].

Biodistribution through pharmacokinetics can be exploited for site‐specific accumulation. In tissues with leaky vasculature, the enhanced permeability and retention (EPR) effect drives passive targeting, yielding 5–50‐fold higher local concentrations than in healthy parenchyma [242]. Active targeting via surface‐conjugated ligands (antibodies, peptides, aptamers) further enhances specificity.

Alternatively, image‐guided local delivery via fine‐gauge needles or via auxiliary channels in endoscopic probes could deposit concentrated sensor suspensions directly at the printing site. Thermo‐reversible hydrogel carriers would retain particles and allow gradual dispersion. An ideal multimodal formulation, yet to be demonstrated, should combine UCNPs, NV nanodiamonds, gold nanoclusters, and oxygen‐sensing polymers for simultaneous thermal, mechanical, photoacoustic, and metabolic readout.

Immediately before fabrication, a short in situ calibration (∼ 30–60 s) accommodates inter‐ and intra‐subject optical and mechanical variability. A low‐power continuous‐wave probe beam applies a controlled 2
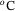
–5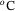 thermal perturbation while the multimodal sensor stream is recorded, and a Gaussian‐process regressor learns the patient‐specific mapping from sensor readings to tissue thermal state. Such personalized calibration is expected to approach the sub‐degree precision intrinsic to the underlying UCNP [161] and OCT readouts, an order of magnitude tighter than fixed and population‐averaged calibrations. The perturbation is reversible and remains below the polymerization threshold, so the baseline is established without committing fabrication energy. The resulting model can then seed an object‐space optimization of the initial exposure plan, in the spirit of dose‐optimization methods developed for volumetric additive manufacturing [65], so that fabrication begins from a plan matched to the local optical and thermal environment rather than from a population‐averaged default.

### 5.2 Real‐Time Control During Printing

The choice of sensing channel during fabrication is constrained by optical accessibility. For superficial or surgically exposed tissue (skin, cornea, open surgical fields), direct optical methods retain full capability while for deep intravital applications through intact tissue, only photoacoustic detection, magnetic resonance thermometry, or fiber‐coupled implanted sensors remain viable.

At the voxel timescale (0.1–100 ms), UCNP ratiometric thermometry serves as the primary sensing channel, providing volumetric thermal mapping suitable for voxel‐level control. The two thermally coupled upconverted bands are acquired with a single hyperspectral or multispectral camera for moderate‐speed monitoring. For kHz‐rate acquisition during pulse‐train modulation, a dual‐camera configuration with narrow bandpass filters provides simultaneous registration of both bands. Compressed sensing exploiting sparsity in temperature gradient fields is applied, minimizing 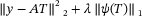, where y is measurement vector, A the sensing matrix, and ψ a sparsifying transform. Field programmable gate arrays (FPGAs) implementations of this class of solver achieve sub‐millisecond latency. For applications demanding ultimate precision at optically accessible sites, NV nanodiamond thermometry provides a complementary sub‐millikelvin channel through continuous ODMR interrogation. Instrumentation complexity currently restricts deployment of ODMR monitoring to single‐point rather than to full‐field imaging.

Phase‐sensitive OCT remains the primary platform for simultaneous thermal and mechanical characterization at the voxel and structure levels, with thermo‐optic phase detection achieving sub‐degree temperature resolution alongside nanometer‐scale displacement sensitivity and elastography tracking of shear‐wave propagation (*v_s_* ≈  1 − 10 m · s^−1^ in soft tissue). Beyond the 1–2 mm penetration limit of OCT, the photoacoustic channel thermometry takes over. Each printing pulse generates a thermoelastic pressure transient whose amplitude scales linearly with temperature through the Grüneisen parameter, enabling thermal monitoring at depths approaching 1 cm with sub‐degree absolute accuracy demonstrated in vivo at 0.1
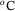
–1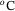.

The acoustic channel reports two distinct phenomena that can be exploited for independent readout. Thermal expansion at each pulse produces a synchronous, broadband (MHz‐GHz) photoacoustic pulse, whereas polymerization‐induced shrinkage generates lower‐frequency (kHz‐MHz) acoustic emission events on the millisecond timescale [243]. The two are separable by timing and frequency content, enabling joint readout of local heating and polymerization completion [244].

### 5.3 Post‐Printing Validation and Longitudinal Monitoring

Once fabrication is complete, the sensing task shifts from millisecond feedback to longitudinal surveillance over hours to months, tracking how the printed structure and the host tissue co‐evolve. The relevant questions to ask are whether the structure retains its intended geometry and mechanics, whether the embedded sensors remain safe and functional, and how the host remodels around the implant. The immediate post‐printing window (the first hour) is monitored through cooling kinetics analysis, UCNP thermometry for superficial constructs and photoacoustic thermometry for deep implants. Deviations from the expected exponential thermal decay carry physiological information. Regions cooling faster than predicted indicate robust local perfusion, while areas of prolonged hyperthermia suggest compromised blood flow and warrant intervention before secondary thermal damage develops.

Structural validation via OCT runs in parallel with thermal monitoring during this window. Phase‐sensitive detection verifies polymerization completeness through refractive index changes, while OCT elastography maps the mechanical contrast between the printed construct and surrounding native tissue. Serial volumetric imaging over the first hour reveals any delayed structural changes (shrinkage, delamination, void formation) requiring intervention. For deep tissue implants beyond optical access, photoacoustic imaging provides the primary post‐printing surveillance channel, tracking both thermal recovery and structural integration through acoustic contrast.

The permanent embedding of thermometric nanoparticles raises safety considerations that must be resolved for clinical translation. Surface‐functionalized UCNPs (PEG, silica, or phospholipid coatings) show no overt toxicity over 90 days in animal models, with retention concentrated in the reticuloendothelial system [245, 246]. However, poorly shielded particles can undergo dissolution with lanthanide ion leakage, potentially causing localized cytotoxicity [247, 248]. Three strategies address this concern: (i) robust protective coatings (phospholipid bilayers, dense silica shells) that prevent degradation over clinically relevant timescales; (ii) biodegradable sensor particles that maintain thermometric function during the critical printing/healing window (hours to days) then safely dissolve and clear [249]; (iii) particle‐free approaches where sensors remain external (fiber‐coupled probes, photoacoustic imaging) rather than embedded.

Short‐term biological response (hours to days) is read through complementary chemical channels. NV nanodiamond *T*_1_ relaxometry tracks local radical concentrations through paramagnetic noise that unpaired electron spins impose on the NV center [250], providing a real‐time oxidative‐stress channel that is distinct from thermal sensing [251, 252]. UCNP emission ratios at appropriate wavelengths additionally report local pH [253, 254] through environmental quenching of selected transitions.

Long‐term monitoring (months to years) exploits photostability of these sensors, which neither bleach nor degrade under repeated interrogation. Periodic low‐power thermal challenges reveal perfusion development through the temperature‐clearance time constant, which shortens from tens of seconds in poorly perfused constructs to a few seconds in well‐vascularized tissue as convective heat transport develops. Continuous data from these sensors could even feed a patient‐specific digital twin of the implanted region, forecasting structural and physiological evolution and enabling proactive intervention before late necrosis or implant failure manifests clinically.

### 5.4 Applications and Emerging Possibilities

The applications below are organized by technical readiness, from the most tractable near‐term target through longer‐term and exploratory directions.

#### 5.4.1 Near‐Term: Ophthalmic Fabrication

Ophthalmic surgery [255, 256] is by far the most immediate setting for closed‐loop intravital photofabrication. The cornea is avascular, transparent, and surgically accessible from the front, making thermal modeling tractable and granting unobstructed optical access for every sensing modality discussed in §3. These properties remove, in a single tissue, most of the obstacles that complicate deeper targets: light penetration, sensor delivery, and thermal confounding from perfusion are all minimized. In situ correction of refractive errors by writing gradient‐index structures, with simultaneous real‐time monitoring of optical aberrations through adaptive‐optics ophthalmoscopy, is the most realistic first demonstration of the full closed‐loop architecture. Because both the fabrication and the primary sensing (OCT phase and elastography) are already established in the eye, the principal remaining step is their integration into a single feedback loop rather than the development of any new physical capability.

#### 5.4.2 Near‐Term to Mid‐Term: Mechanically and Thermally Demanding Targets

Two further targets are technically plausible in the near‐to‐mid‐term but impose stricter control requirements. Cardiac repair after infarction presents a mechanical challenge. Closed‐loop control could create mechanically graded patches that bridge the abrupt stiffness contrast between fibrotic scar and compliant myocardium, suppressing the interfacial stress concentrations identified in §2.2. Closed‐loop could be implemented through real‐time elastography that maps the gradient, and thermal sensing that helps prevent collagen‐denaturation [257, 258, 259]. Neural interfaces are the most thermally demanding. Polymerizing conductive polymers within brain tissue would require sub‐degree thermal control, since neurons show functional deficits at Δ*T* as small as 2°C–3 °C [260, 261], a regime that only continuous closed‐loop monitoring can hold despite local variations in perfusion and metabolism.

#### 5.4.3 Boundary of Feasibility: Bone and Cartilage

Bone and cartilage replacement mark the current boundary of feasibility and illustrate where intravital photofabrication does not yet reach. Ex vivo two‐photon polymerization of bone‐mimetic scaffolds with submicron resolution and osteogenic biocompatibility is well established [262]. Yet no work has demonstrated intravital fabrication of bone‐replacement scaffolds in living tissue. Conventional methods [263] require temperatures of hundreds to thousands of degrees and/or inert atmospheres incompatible with living hosts. Articular cartilage repair faces the analogous gap: laser‐based intravital fabrication cannot match the throughput of conventional bioprinting but offers what extrusion methods cannot.

Across these targets, recent demonstrations of machine‐learning parameter prediction and deep‐learning visual inspection in multiphoton polymerization [264, 265] indicate that the computational components of the closed‐loop architecture are already maturing in adjacent settings and can be transferred to the intravital regime once validated sensing datasets exist.

#### 5.4.4 Longer‐Term and Exploratory Directions

The following directions are more speculative, and this manuscript presents them explicitly as longer‐term possibilities rather than near‐term prospects. In 4D bioprinting [266], stimuli‐responsive monomers form networks that evolve in response to biocompatible cues (enzymes, glucose, signaling molecules) [267]. Closed‐loop control during printing could pre‐program such responses from the measured local state, with stiffness measurements informing crosslinking density so that structures mature in synchrony with tissue remodeling. The broader field of stimuli‐responsive and 4D‐printed materials has advanced rapidly across diverse functionalities and applications, and real‐time sensing of the evolving material state would provide a route for controlling these responsive transformations [268, 269, 270] during rather than after fabrication, a capability that current 4D‐printing approaches largely lack. Immunomodulatory printing would use real‐time sensing of oxidative stress (NV relaxometry) and pH (UCNP ratiometric emission) to guide local deposition of anti‐inflammatory agents [271]. Tumor‐responsive therapy could exploit printing drug‐delivery depots whose release kinetics adapt to the sensed microenvironment [272, 273]. These directions are included to indicate the reach of the closed‐loop concept. Their realization depends on advances in responsive materials and multiplexed sensing in the coming years.

## 6 Challenges, Limitations and Future Perspective

The architecture proposed in §3 to §5 is based on techniques that have already been demonstrated (Table 1). The challenge will be to operate these techniques together, in living tissue, at the rates the loops demand. The obstacles fall into two classes. The first is component‐level. Sensor performance degrades in the biological environment through background emission, non‐uniform distribution, calibration drift, and host response. The second is integration, and it determines whether the loop closes at all. This requires an in situ readout of conversion that separates the chemical from the thermal contribution to the measured phase (§4.1), a control loop validated in living tissue rather than in phantoms, and the labeled datasets needed to train and certify it.

**TABLE 1:** Technology readiness of the closed‐loop intravital photofabrication architecture. Each element listed has been experimentally demonstrated; the table records the demonstration context, whether in vivo in living tissue, ex vivo or in vitro outside a living host. Every element has been shown individually, yet none has been operated as a real‐time feedback element within an intravital fabrication loop. The sensing platforms report without actuating; the control methods have been validated in laser processing or volumetric additive manufacturing rather than in living tissue and intravital photofabrication.

Architecture element	Demonstration context
***Fabrication***	
Two‐photon polymerization in living tissue	In vivo mouse dermis, skeletal muscle, and brain; transcutaneous and intracellular writing [4]
***Primary sensing***	
UCNP luminescence thermometry	In situ during direct laser writing [76, 167]
OCT thermo‐optic thermometry	In vivo retina [134, 146]
Phase‐sensitive OCT elastography	In vivo imaging; nanometer‐scale displacement sensitivity [149]
***Complementary sensing***	
Photoacoustic thermometry	In vivo sub‐degree accuracy at depths >1 cm [197]
Brillouin microscopy	Ex vivo */* in vitro millisecond voxel acquisition via VIPA and stimulated configurations [169, 184, 185]
NV‐diamond thermometry and relaxometry	In vivo cellular; ∼30 mK at 1 kHz^180^
Acoustic emission monitoring	Ex vivo composites and non‐destructive testing [188]
***Multimodal integration***	
Co‐integrated OCT and photoacoustic imaging	In vivo mouse ear; single megahertz supercontinuum laser [204]
Optical sensing co‐registered with a therapeutic laser	Ex vivo single double‐clad fiber, coagulation‐depth feedback [206]
***Control***	
Model‐predictive thermal control	Laser additive manufacturing, melt‐pool temperature control with defect mitigation [233]
Object‐space dose optimization from a known material model	Volumetric additive manufacturing; ex vivo resin [65]
Machine‐learning correction of fabrication deviation	Ex vivo direct laser writing; up to 90% reduction in geometric deviation [274, 275, 276, 277]

### 6.1 Technical Barriers

Signal‐to‐background ratio in biological tissues presents immediate challenges for sensing [278]. Endogenous fluorophores (NADH, flavins, collagen, elastin) generate broadband emission that can overwhelm thermal sensor signals, particularly under two‐photon excitation. Time‐gated detection [279, 280], exploiting microsecond UCNP lifetimes against nanosecond autofluorescence, suppresses the short‐lived background almost entirely and improves signal‐to‐background ratios by one to two orders of magnitude in vivo, but requires sophisticated instrumentation (pulsed excitation, fast‐gating image intensifiers, precise synchronization). Spectral unmixing algorithms model measured spectra as linear combinations of basis spectra [281, 282] (*E_i_* (λ)) and noise (*N*(λ)), as *S* (λ) = Σ_*i*_  *a_i_E_i_* (λ) + *N*(λ), and recover the abundances *a_i_* by least‐squares inversion. Machine‐learning methods extend this to blind or nonlinear unmixing where the basis spectra are unknown, or in the case of nonlinear responses. Real‐time unmixing of full hyperspectral volumes at video rate benefits from dedicated hardware acceleration (FPGA or GPU).

Achieving a uniform sensing particles distribution remains challenging. The blood‐brain barrier excludes most nanoparticles from central nervous system applications. The reticulo‐endothelial system rapidly sequesters particles in the liver and spleen [283] and preferential accumulation in perivascular spaces can leave parenchymal regions under‐sampled. Advanced functionalization strategies continue to evolve. Cell‐penetrating peptides facilitate transcytosis and biomimetic red blood cell membrane coatings exploit natural trafficking pathways. Physical approaches, like magnetic manipulation helps concentrate superparamagnetic particles post‐injection [284].

Calibration drift in biological environments poses a further challenge. Local pH fluctuations alter luminescence quantum yields by tens of percent across the physiological range [285]. Protein adsorption modifies surface properties and energy transfer rates [286]. Enzymatic degradation of organic components changes sensor characteristics. As a countermeasure, ratiometric sensing provides partial immunity, and in situ recalibration through periodic controlled heating preserves accuracy.

Biological responses to sensor materials may introduce additional variability and sensitivity. Even nominally biocompatible materials trigger some foreign‐body response, like protein adsorption, complement activation, recruitment of inflammatory cells, fibrous encapsulation. All these modify the properties of the micro‐environment and the behavior of sensors. Immunomodulatory strategies (CD47 “don't eat me” peptides [287], anti‐inflammatory agent incorporation) attempt to minimize responses. However, optimal strategies likely involve hierarchical protection: immediate immunosuppression during printing when precise sensing is critical, followed by controlled integration.

### 6.2 Regulatory and Ethical Considerations

Validation of the proposed closed‐loop systems requires the demonstration of their safety and efficacy across an enormous parameter space, type and state of tissue, sensor inputs and control outputs. Current regulatory frameworks struggle with adaptive devices that exploit machine learning rather than explicit programming [288]. International consortia are establishing standardized testing protocols for nanothermometer biocompatibility, sensing accuracy, and long‐term stability.

Liability and accountability become complex when autonomous systems make microsecond decisions affecting patient outcomes. New legal frameworks must address responsibility distribution between device manufacturers, algorithm developers, and clinicians. The use of sensors implanted in humans, even if available within a restricted network, raises privacy concerns about biological surveillance and data ownership.

The high cost of such sophisticated systems may potentially limit the access to advanced regenerative therapies, particularly for low‐income populations and low‐income countries, and raises equity concerns. In addition, and over a longer term, the potential for enhancement beyond restoration (printing structures exceeding normal biological capabilities) opens even more complex ethical territories about human modification and fairness.

### 6.3 Future Perspective

To foster implementation, this manuscript groups future perspectives by plausibility. Some are near‐term extensions of existing capability, others are longer‐term prospects dependent on advances in adjacent fields, and a few remain exploratory. None is yet demonstrated in the intravital setting.

#### 6.3.1 Near‐Term Extension

New sensors are continuously developed and existing ones improved. Two‐dimensional materials represent a promising sensing frontier. Graphene's Raman G‐peak shifts linearly with temperature (−0.016 cm K^−1^) [289], enabling label‐free measurement with diffraction‐limited resolution. Monolayer MoS_2_ photoluminescence exhibits strong thermal quenching with sensitivity exceeding 1% per Kelvin [290]. Their atomic thickness (<1 nm) enables insertion between cells without disrupting tissue architecture, providing thermal information from previously inaccessible locations. While pristine 2D materials exhibit dose‐dependent cytotoxicity, surface functionalization, particularly PEGylation, significantly improves biocompatibility and enable neural tissue integration [291, 292].

#### 6.3.2 Longer‐Term Prospect

Quantum sensing continues advancing toward practical implementation. Silicon vacancy centers in silicon carbide offer biocompatible hosts, near‐infrared emission reducing tissue absorption, and coherence times exceeding milliseconds at room temperature [293]. Nuclear spin sensors push sensitivity toward fundamental limits through chemical shift changes with zepto‐Kelvin sensitivity [294]. Integration with nanophotonic cavities enhances collection efficiency, suggesting possibilities for massively parallel quantum sensing through on‐chip optical networks.

Artificial intelligence transforms raw data into “actionable intelligence”. Deep reinforcement learning discovers optimal strategies through experience, learning complex mappings that maximize long‐term outcomes rather than merely preventing immediate damage. Digital twins of specific patients constructed from preoperative imaging, and fed by post‐operative monitoring, enables personalized optimization before intervention [295]. Federated learning enables models to improve from distributed experience without sharing sensitive patient data.

#### 6.3.3 Exploratory

Biological engineering approaches would merge synthetic biology with thermal sensing. Engineered bacteria expressing temperature‐sensitive fluorescent proteins could in principle report temperature through ratiometric readouts, offering the more speculative prospect of self‐replicating sensors within a printed construct [296, 297]. Synthetic gene circuits implementing thermal logic, such as heat‐shock promoter switches activating at 40°C–45°C with >200‐fold dynamic range, could trigger therapeutic responses including drug release when temperature exceeds defined thresholds [298, 299]. These directions remain conceptual and would require substantial advances in biocontainment and regulatory acceptance before printed constructs with embedded biological intelligence could be contemplated in a living host.

## 7 Conclusion

The transition from blind to guided intravital printing marks a fundamental shift in how we approach biological fabrication. By operating at the femtoliter scale where laser energy, tissue mechanics, and cellular fate converge, closed‐loop control transforms heterogeneity from being an obstacle to an information carrier. Each printed voxel becomes a sensor‐actuator unit engaged in continuous dialogue with the surrounding tissue, adapting parameters in real‐time to achieve optimal integration. This continuous dialogue is precisely what distinguishes the emerging paradigm discussed in this manuscript from conventional automation. It elevates the fabrication system into a “digital twin” of the biological target, a virtual replica of the printing volume that is continuously updated by sensor data and used to forecast, optimize, and correct the next control action [233].

This vision requires convergence of multiple technologies: ultrafast lasers with agile control, multimodal sensing with cellular resolution, and sophisticated algorithms capable of fast (milli to microsecond) decision‐making. As discussed in Table 1, we are already witnessing the possibility of adaptive feedforward with supervisory feedback. Encouragingly, the pieces for a real closed‐loop architecture are also falling into place. Recent work has demonstrated machine‐learning‐based pre‐correction of fabrication deviations in direct laser writing [276], closed‐loop control of two‐photon‐fabricated MEMS scanners with integrated Hall sensors [277], and deep‐learning‐based correction of direct laser writing alignment errors with up to 90% reduction in geometric deviation [275]. Together with advances in photonic integration, adaptive optics, and quantum‐sensor miniaturization, these results bring closed‐loop intravital photofabrication within technical reach, though no integrated closed‐loop demonstration in living tissue has yet been reported.

The implications extend beyond technical achievement. Closed‐loop control represents a new philosophy of biomedical intervention, potentially embracing biological complexity rather than attempting to suppress it. Instead of forcing tissues to conform to our designs, we learn to speak their language, adapting our tools to their needs. In this sense, the digital twin is not merely a computational convenience but a conceptual reframing. The patient becomes part of the manufacturing system, and the manufacturing system becomes accountable to the patient. This collaborative approach may ultimately prove more powerful than any attempt at absolute control.

As we stand at this technological threshold, the path forward is clear: develop the sensing modalities to read tissue state at relevant scales, create the algorithms to interpret this multidimensional information, and build the control systems to respond appropriately within therapeutic timescales. The challenge is formidable, but the reward, truly adaptive intravital photofabrication that works with rather than against living systems, justifies the effort.

## Nomenclature

SymbolDefinition: DefinitionUnits

### Optical excitation and fabrication

I
: Optical intensity at the focus, W cm^−^^2^
*I*_th_
: Polymerization threshold intensity, W cm^−^^2^
*I*_0_
: Incident (surface) intensity, W cm^−^^2^
*σ*_2_
: Two‐photon absorption cross‐section, GM
*Φ*_R_
: Radical quantum yield, —
*G*_R_
: Radical generation rate, m^−^^3^ s^−^^1^
NA
: Numerical aperture, —
*w*_0_
: Beam waist radius, µm
*v*_scan_
: Scan velocity, µm s^−^^1^
*D*_acc_
: Accumulated dose, nJ
τ
: Dwell / pulse dwell time, s
*V*_voxel_
: Voxel volume, fL
λ
: Wavelength, nm
C_A_
: Spectral correction factor, —

### Thermal field and damage

*T*
: Temperature, field T(r,t), °C / K
*ρ*
: Tissue mass density, kg m^−^^3^
*c*_p_
: Specific heat capacity, J kg^−^^1^ K^−^^1^
*k*
: Thermal conductivity, W m^−^^1^ K^−^^1^
*µ*_a_
: Optical absorption coefficient, cm^−^^1^
*F*
: Optical fluence, J cm^−^^2^
*C*_heat_
: Heat capacity of heated volume, J K^−^^1^
*τ*_diff_
: Thermal diffusion time, s
Ω: Arrhenius thermal‐damage integral, —
*A*
: Arrhenius frequency factor, s^−^^1^
*E*_a_
: Activation energy, kJ mol^−^^1^
*R*
: Universal gas constant (Eq. 2),J mol^−^^1^ K^−^^1^
*k*_B_
: Boltzmann constant, J K^−^^1^
*H_p_*
: Reaction enthalpy, J

### Mechanical properties

*E*(*D)*
: Elastic modulus, Pa
*D*
: Degree of conversion, —
*m*
: Network power‐law exponent, —
*ε*_max_
: Maximum volumetric strain, —
*σ*_shrink_
: Shrinkage‐induced stress, Pa
ν: Poisson ration, —
*M*
: Longitudinal (Brillouin) modulus, GPa
*G*
: Shear modulus, kPa
*v*_s_
: Shear‐wave velocity, m s^−^^1^

### Sensing observables

*n*
: Refractive index, —
d*n*/d*T*
: Thermo‐optic coefficient K^−^^1^
Δ*φ*
: Thermo‐optic phase shift (OCT), rad
*k_O2_, k_d_*
: Rate of Oxygen inhibition, and radical loss, M^−1^ s^−1^, s^−1^
*R*
: UCNP emission ratio I_5__2__5_/I_5__4__5_, —
*S*_a_
: Absolute thermal sensitivity, K^−^^1^
Δ*E*
: Energy gap (coupled levels / chromophore), cm^−^^1^
*Γ*
: Grüneisen parameter (photoacoustic), —
*β*
: Thermal expansion coefficient, K^−^^1^
*p*_0_
: Photoacoustic initial pressure, Pa
*ν*_B_
: Brillouin frequency shift, GHz
*θ*
: Scattering angle, Brillouin), rad
*D*
: NV zero‐field splitting (≈2.87 GHz), GHz
d*D*/d*T*
: NV temperature shift coefficient, kHz K^−^^1^
δ*T*_min_
: Minimum detectable temperature change, K
𝒞: ODMR contrast, —
*T*_2_*: NV spin dephasing time, µs
*t*_meas_
: Total measurement time, s
*N_ph_*
: Photon number, shot‐noise limit), —

### Control and estimation

*N*
: MPC prediction horizon, —
*P*(*k)*
: Laser power (control input) at step k, W
*T*_ref_
: Reference temperature trajectory, °C
Q: MPC state‐error weight matrix, —
R_u_
: MPC input‐effort weight matrix, —
M
: Reduced‐order thermal model operator, —
*ξ*
: Tissue photothermal coefficient vector, —
$\hat{\xi }$
: Estimated photothermal coefficient, —
K(*k)*
: Observer / Kalman gain, —
*u*(*k)*
: General control input, —
*u*_probing_
: Persistent‐excitation dither signal, —
*a*_i_, *φ*_i_
: POD modal coefficients / spatial modes, —

### Reproducibility and fusion

*P*_s_
: Joint probability of fabrication *success*, —
*δ*_i_, *σ*_i_
: Tolerance and standard deviation, *parameter* i, —
*X*, *Z*
: Tissue state; sensor measurements (*Bayesian*), —
*h*_i_
: Observation model of sensor i, —
**Notation conventions**
: Scalar physical variables are set in italic; vectors and matrices in upright bold; units, chemical species, and mathematical operators in upright roman . $\hat{\xi }$ denotes an estimate. Where a single letter carries more than one meaning, the context (and subscript) is unambiguous: *R* denotes the UCNP emission ratio except in the Arrhenius integral (gas constant) and the MPC weight *R*_u_; *D* denotes degree of conversion except for the NV zero‐field splitting.

## Author Contributions

**Amirbahador Zeynali**: conceptualization, investigation, writing – original draft, visualization, writing – review and editing, project administration, supervision, resources, funding acquisition, methodology. **Mangirdas Malinauskas**: validation, formal analysis, conceptualization, writing – review and editing, funding acquisition. **Giuseppe Chirico**: conceptualization, validation, writing – review and editing, formal analysis, methodology, supervision.

## Funding

This work was supported by the European Union's Horizon 2020 research and innovation program under the Marie Skłodowska‐Curie grant agreement No. 101110870. This research was carried out in the framework of the ‘Universities’ Excellence Initiative'program by the Ministry of Education, Science and Sports of the Republic of Lithuania under the agreement with the Research Council of Lithuania (project no. S‐A‐UEI‐23‐6).

## Conflicts of Interest

The authors declare no conflicts of interest.

## Data Availability

Data sharing not applicable to this article as no datasets were generated or analysed during the current study.
